# Gastric bypass surgery in a rat model alters the community structure and functional composition of the intestinal microbiota independently of weight loss

**DOI:** 10.1186/s40168-020-0788-1

**Published:** 2020-02-07

**Authors:** Sven-Bastiaan Haange, Nico Jehmlich, Ute Krügel, Constantin Hintschich, Dorothee Wehrmann, Mohammed Hankir, Florian Seyfried, Jean Froment, Thomas Hübschmann, Susann Müller, Dirk K. Wissenbach, Kang Kang, Christian Buettner, Gianni Panagiotou, Matthias Noll, Ulrike Rolle-Kampczyk, Wiebke Fenske, Martin von Bergen

**Affiliations:** 1 Department of Molecular Systems Biology, Helmholtz Centre for Environmental Research-UFZ, Leipzig, Germany; 2 Institute of Biochemistry, Faculty of Life Sciences, University of Leipzig, Leipzig, Germany; 3 Rudolf Boehm Institute of Pharmacology and Toxicology, Medical Faculty, University of Leipzig, Leipzig, Germany; 4 Neuroendocrine Regulation of Energy Homeostasis Group, IFB Adiposity Diseases, Leipzig, Germany; 5 Department of General, Visceral, Vascular and Pediatric Surgery, Wuerzburg University Hospital, Wuerzburg, Germany; 6 Department of Environmental Microbiology, Helmholtz Centre for Environmental Research-UFZ, Leipzig, Germany; 7 Systems Biology and Bioinformatics Group, School of Biological Sciences, The University of Hong Kong, Hong Kong SAR, China; 8 Department of Microbiology, Li Ka Shing Faculty of Medicine, The University of Hong Kong, Hong Kong SAR, China; 9 Institute for Bioanalysis, Faculty of Applied Sciences, Coburg University of Applied Sciences and Arts, Coburg, Germany; 10 Leibniz Institute for Natural Product Research and Infection Biology, Hans Knoll Institute, Jena, Germany; 11 Current address: Department of Experimental Surgery, Wuerzburg University Hospital, Wuerzburg, Germany; 12 Current address: Institute of Forensic Medicine, Jena University Hospital, Jena, Germany

## Abstract

**Background:**

Roux-en-Y gastric bypass (RYGB) surgery is a last-resort treatment to induce substantial and sustained weight loss in cases of severe obesity. This anatomical rearrangement affects the intestinal microbiota, but so far, little information is available on how it interferes with microbial functionality and microbial-host interactions independently of weight loss.

**Methods:**

A rat model was employed where the RYGB-surgery cohort is compared to sham-operated controls which were kept at a matched body weight by food restriction. We investigated the microbial taxonomy and functional activity using 16S rRNA amplicon gene sequencing, metaproteomics, and metabolomics on samples collected from theileum, the cecum, and the colon, and separately analysed the lumen and mucus-associated microbiota.

**Results:**

Altered gut architecture in RYGB increased the relative occurrence of *Actinobacteria*, especially *Bifidobacteriaceae* and *Proteobacteria*, while in general, *Firmicutes* were decreased although *Streptococcaceae* and *Clostridium perfringens* were observed at relative higher abundances independent of weight loss. A decrease of conjugated and secondary bile acids was observed in the RYGB-gut lumen. The arginine biosynthesis pathway in the microbiota was altered, as indicated by the changes in the abundance of upstream metabolites and enzymes, resulting in lower levels of arginine and higher levels of aspartate in the colon after RYGB.

**Conclusion:**

The anatomical rearrangement in RYGB affects microbiota composition and functionality as well as changes in amino acid and bile acid metabolism independently of weight loss. The shift in the taxonomic structure of the microbiota after RYGB may be mediated by the resulting change in the composition of the bile acid pool in the gut and by changes in the composition of nutrients in the gut.

Video abstract.

## Introduction

**Fig. 1:**
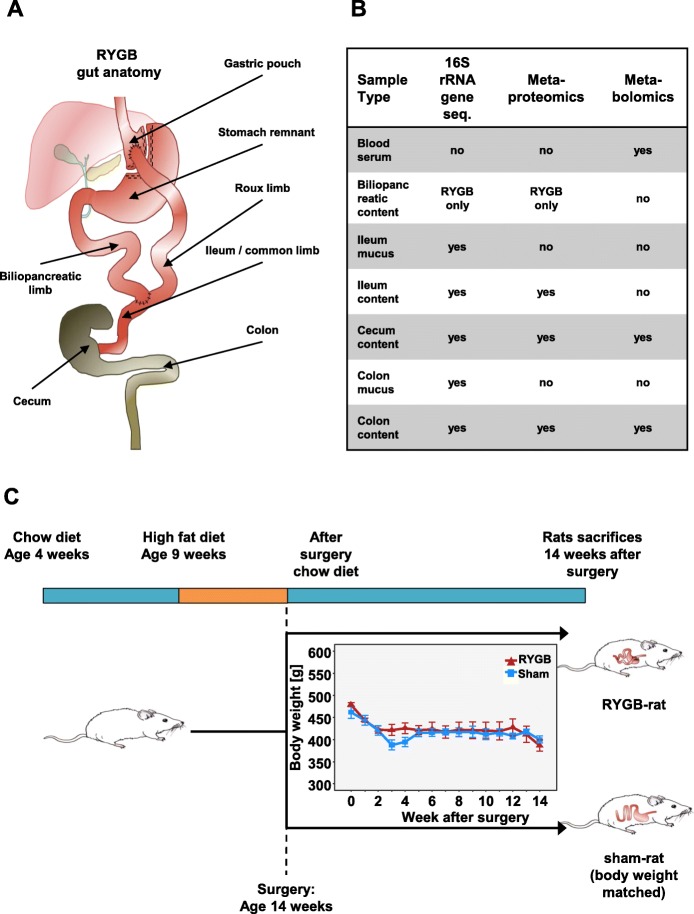
Experimental setup with anatomy of the gut after RYGB (**a**), analysis methods used on samples (**b**), and time line of diet and postoperative body weight development (**c**) (error bars are SEM)

Roux-en-Y gastric bypass (RYGB) surgery is an effective long-term treatment strategy for weight loss and hyperglycemia in patients with obesity and type 2 diabetes [1–4]. The procedure has emerged as a research model to understand the pathophysiological mechanisms underlying both obesity and its associated complications [5]. In RYGB, the anatomy of the gastrointestinal tract is systematically altered (Fig. 1a), which significantly modifies the intestinal environment and has the potential to change and disrupt the gastrointestinal microbiota [6–8]. Re-routing the duodenum into the distal jejunum results in altered bile flow and modulation of enteric and adipose hormones (Fig. 1a) [9–11].

The commensal intestinal microbiota is crucial for the degradation of otherwise non-digestible compounds into absorbable metabolites and the synthesis of essential vitamins [12–15]. The microbiota is a key player in the regulation of immune responses to pathogenic species [16]. RYGB drastically changes the amount and composition of nutrients available to the intestinal microbiota [7]. Currently, there is a limited number of studies describing RYGB changes in respect to the intestinal microbial community. These studies mainly investigated in taxonomic distribution using next-generation sequencing of murine fecal samples [17] or focused on weight-loss effects [18]. Expectedly, one study could support the claim that changes in the gut microbiota contribute to reduced host weight and adiposity after RYGB surgery [19].

Metabolomics is currently considered as the most appropriate omics technology to investigate complex, polygenic, and multifactorial diseases with a strong multisystemic metabolic nature which have been successfully used to investigate functional changes after RYGB [20–22]. Recently, several studies have shown that metaproteomics which involves the high-throughput characterisation of the entire constituent profile of microbial proteins provides promising insights of functional aspects in microbiome research [23–25].

Findings in the microbial community associated with the rearrangement of the gastro-intestinal tract after RYGB surgery and identifying whether these modifications are the cause or consequence of weight loss will be of importance, because it will greatly contribute to the discovery of future therapies for adult patients with overweight or obesity while assessing the risk of long-term side effects.

Alteration to the gastro-intestinal tract introduced by RYGB-surgery restructure the microbiota on a functional and taxonomic level including changes in abundance of proteins and metabolites. In this study, we used an RYGB rat model to study the RYGB-surgery-specific and body weight-loss-independent effect on the microbiota. Thus, the rats received postoperatively a well-tolerable standard chow. Importantly, the diet was applied in RYGB- as well as in Sham-operated body weight-matched control animals in order to control for confounders secondary to nutrient composition. We applied 16S rRNA amplicon gene sequencing, metaproteomics, and metabolomics to investigate the microbiota to highlight taxonomic and more importantly the functional changes introduced by the RYGB surgery.

## Material and methods

For a detailed description of methods, see Additional file 1.

### Animals

Male Wistar rats (RjHan: WI, outbred, Janvier, Le Genest-Saint-Isle, France) were used for our studies. All experiments and animal care were approved by the Institutional Animal Care and Use Committee at the University of Leipzig with the permission of the local government of Saxony (Regional Administrative Authority Leipzig, TVV 63/13, Germany). Feed and water were provided ad libitum unless otherwise indicated. Rats were 9 weeks old and initially weighed approximately 350 g. Diet-induced obesity (DIO) was achieved by feeding animals for 5 weeks with a high-fat diet (HFD), which provides 58% of total energy as fat, 25.5% as carbohydrate, and 16.5% as protein (EF D12331, Ssniff GmbH, Soest, Germany). After surgeries, animals were individually housed (Fig. 1).

### Abdominal surgery and postoperative care

All surgical procedures were carried out after an overnight fast. The RYGB procedure was performed according to an established protocol [26, 27].

Postoperatively, all animals were given standard laboratory chow mixed with water (wet diet) for 48 h before being returned onto solid standard laboratory chow. Sham-operated animals were kept at a body weight matched to that of RYGB rats (Sham-BWM group) by restricting the amount of feed. The amount of feed given was calibrated daily [28]. After a postoperative 2-week stabilisation period, body weight and feed intake were recorded daily.

### Microbiome sampling

Animals were sacrificed 3 months after the operation [29]. The gastrointestinal tract was rapidly removed and cut into segments. For microbiome sampling, the last 3 cm of the ileum, cecum, and distal colon (3 cm) were cut longitudinally and opened up as previously described [30]. The lumen contents were removed and shock frozen in liquid nitrogen. In RYGB, the contents of the last 2 cm of the biliopancreatic limb were also sampled. Ileum and colon mucus were sampled as previously described [30] and stored at − 20 °C.

### Flow cytometry, cell sorting, and further analysis of cecum samples

See Additional file 1.

### Metabolite extraction from samples

Metabolites were extracted from cecum and distal colon content samples by adding 5 μL H_2_O/acetonitrile (1:1, v:v) per 1 mg of the sample then homogenising with a tissue slicer (10 min, 30 Hz, 4 steel balls). This was followed by sonication (5 min). Samples were centrifuged (14,000*g* at 2 min), and the supernatant was aliquoted for targeted and untargeted metabolomics and kept at − 80 °C. Serum samples were frozen and stored without preparation at − 80 °C.

### Protein and DNA extraction

Cell lysis followed by protein and DNA extraction was done as previously described [31]. Samples from the biliopancreatic limb lumen content (*n* = 4), ileum mucus (*n* = 5), ileum lumen content (*n* = 5), cecum lumen content (*n* = 5), colon mucus (*n* = 5), and colon lumen content (*n* = 5) each from RYGB rats and Sham-BWM rats were randomly chosen for bacteria lysis. For content samples, approximately 0.5 g of sample was chosen, while the entire content of the mucus samples was used. These samples were thawed and resuspended in 1 mL lysis buffer (50 mM Tris, 5 mM EDTA, 0.4% SDS, 50 mM NaCl, 1 mM PMSF, pH = 8) and disrupted with a FastPrep (FastPrep-24, MP Biomedicals). Then, samples were heated in a Thermomixer (Thermomixer comfort 5355, Eppendorf) at 60 °C with shaking at 1400 rpm for 15 min. This was followed by sonication using an ultrasound probe (UP50H, Hielscher), and samples were spun at 10,000 rcf at 4 °C for 10 min. Supernatants, containing the DNA and protein content, were kept. The pellets were resuspended in 300 μL of lysis buffer, and cell lysis was repeated. The resulting supernatant of each sample was added to the corresponding supernatant of the first lysis round and frozen at − 20 °C for storage.

Protein extraction and proteolytic cleavage for metaproteomics were done to a modified method [31]. Briefly, 150 μg of protein was precipitated from each lysate (Fig. 1b), separated by SDS-PAGE and further processed by in-gel reduction and alkylation of cysteine residues followed by cleavage of proteins with trypsin, eluting of resulting proteolytic peptides and desalting peptides as previously described [31]. The modification was that whole SDS-PAGE lanes were cut into five separate fractions each and handled separately. Proteolytic peptide lysate was measured using nanoLC-MS/MS for metaproteome analysis (see Additional files 1 and 2).

DNA extraction was done as previously described [31]. Briefly, 260 μL NH_4_ acetate (10 M) was added to 500 μL lysate (Fig. 1b), and samples were incubated on ice and centrifuged. Equal volume of ultrapure isopropanol was added to the supernatant, mixed thoroughly, and incubated on ice for 30 min. Samples were centrifuged, and pellets were washed with 70% ethanol, vacuum-dried, and resolved overnight in TE buffer (1 mM EDTA, 10 mM Tris, pH 8). DNA was purified, and proteins removed using the QIAamp DNA Mini Kit (Qiagen, Valencia, CA USA) according to the manufacturer’s instructions. Purified DNA samples were sent to Molecular Research DNA (MR DNA, Shallowater, TX, USA) for library preparation and sequencing.

### Meta-omics analysis

16S rRNA gene profiling, metaproteomics, and metabolomics were performed on different samples (Fig. 1b). Detailed descriptions of the omics methods are provided in Additional files 1 and 2.

16S rRNA gene sequencing resulted for the biliopancreatic limb samples in 75,249 ± 5306 reads; for the ileum mucus in 78,921 ± 7843 reads for RYGB and 65,950 ± 12,243 reads for Sham-BWM; and for the ileum lumen content 77,747 ± 7130 reads in RYGB and 77,493 + -2,716 reads for Sham-BWM. In the cecum, amplicon sequencing resulted in 140,779 ± 12,822 reads for RYGB and 89,567 ± 13,794 reads for Sham-BWM. In the colon, in the mucus samples, for RYGB, 65,213 ± 8564 reads, and for Sham-BWM 45,900 ± 5142 reads were detected, whereas in the lumen content, 53,248 ± 3889 reads in RYGB and 50,199 ± 6541 reads for Sham-BWM were observed.

In total, in the biliopancreatic limb samples (*n* = 4), 3199 protein groups were identified, while in the ileum content samples (*n* = 5 for RYGB and for Sham-BWM), a total of 6496 protein groups were identified. In the large intestine, the cecum content (*n* = 5 for RYGB and for Sham-BWM) yielded in total 12,570 protein groups, whereas the colon content (*n* = 5 for RYGB and for Sham-BWM) yielded 8985 protein groups.

For the targeted metabolomics, 207 polar and nonpolar metabolites were measured. These included 20 bile acids, 22 amino acids, 20 amines, 40 acylcarnithines, 89 glycerophospholipid, and 15 sphingomyelins. In addition, sugars were measured as one parameter.

### 16S rRNA gene sequencing data analysis and statistics

The relative number of reads assigned to each of the bacterial taxa in each sample was used for taxonomic analysis. Statistical analysis and data visualisation were performed using R. For OTU-level analysis, the R package *Rhea* was used to normalise data, calculate alpha diversity, and statistical analysis [32]. PD Faith index [33] was calculated using the ape and picante R packages. All *p* values were corrected for multi-testing using Benjamini-Hochberg.

### Metaproteomic data analysis and statistics

PROteomics results Pruning & Homology group ANotation Engine (PROPHANE) was used to assign proteins to their taxonomic and functional groups [34]. For each protein group, the taxonomy annotation was based on the NCBInr protein database using BLASTP v2.2.28+ on all proteins binned to the protein group and only considering hits with an *e* value ≤ 0.01. The functional prediction of protein groups was based on COG database and KOG database using RPSBLAST v2.2.28+ on all proteins from the protein group and only considering hits with an *e* value ≤ 0.001 [34–36].

Transforming, normalisation, and statistical analysis of intensity data from protein groups were performed by R scripts. Briefly, summed intensities were log_10_ transformed and median normalised. Only protein groups identified in at least three biological replicates out of five in both conditions (RYGB and Sham-BWM) were considered for relative quantification. For statistical analysis of fold changes, a two-sided independent Student test was performed. For a protein group to be considered unique for one condition, it had to be identified in at least three replicates of that treatment and in none in the other treatment with *P* calculated using the Wilcoxon rank test. All *P* were corrected for multi-testing using the Benjamini-Hochberg method [37]. The taxonomic analysis of metaproteome data was only applied for taxa which were identified in at least three replicates out of five in one condition. For functional pathway analysis, KEGG [38] as well as the Metacyc [39] was used. Significance for the entire pathways was calculated by the sum of *P* method (sump) [40] implemented in the metap package while the values of the adjusted *P* from the relative number of protein groups involved in the pathway, the unique protein groups, and the LFQ values of the relative quantifiable protein groups were combined.

### Metabolomic data analysis and statistics

For targeted metabolomics, the integrated MetIDQ software (Biocrates, Innsbruck, Austria) streamlines data analysis by automated calculation of metabolite concentrations providing quality measures and quantification [41]. Statistical analysis of metabolite concentrations was performed by two-sided independent Student tests.

The analysis of untargeted metabolomics was done by loading raw data to *XCMS* online [42] to perform the peak picking, grouping of similar peaks, and retention time alignment. Then, only the peaks appearing in at least 80% of the replicates of one condition and above the intensity threshold of 2000 counts were selected for the statistical analysis. Nonparametric multi-dimension scaling (NMDS) of the selected peaks was carried out using the *vegan* package.

## Results

### Changes in animal body weight and host phenotypical parameters

Post-surgery, Sham-BWM animals were kept at similar bodyweight as RYGB animals (Fig. 1c). To confirm the metabolic stability of the host organisms, we performed a targeted analysis of serum metabolites in RYGB and Sham-BWM, while we identified only slightly differences (Additional file 1: Figure S1). Among the 207 measured metabolites, only seven revealed significant (*P* < .05) abundance changes including the bile acid MCA(b) (*P* = .04) and acylcarnitine C3-acylcarnithine (*P* = .0062) with significantly higher abundances in RYGB, whereas glycerophospholipid PC aa C42:2 (*P* = .009) was observed with lower abundance in RYGB. Notably, five sphingomyelins SM (OH) C24:1 (*P* = .014), SM C16:0 (*P* = .0016), SM C16:1 (*P* = .0012), SM C24:0 (*P* = .0274), and SM C26:1 (*P* = .0062) were detected at lower abundances for the RYGB samples.

### Metaproteomics indicates that nutrients less efficiently digested in RYGB

**Fig. 2:**
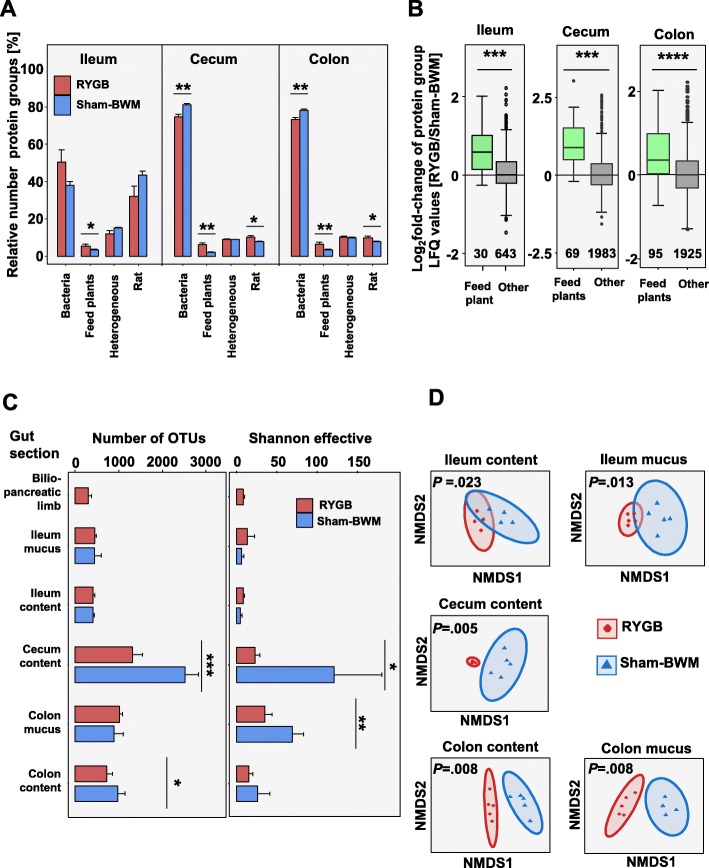
Global view of the gut microbiota (****P < .0001, ***P < .001, **P < .01, *P < .05). **a** Relative number of protein groups identified in the contents of the three gut sections. **b** Boxplot of protein groups which were relatively quantifiable by label-free quantification (LFQ). Number in plot represents the number of protein groups in the boxplot. **c** Alpha diversity of 16S rRNA gene sequencing data at OTU level. Richness based on OTU numbers (left) and Shannon-effective diversity index (right). **d** Beta-diversity of 16S rRNA gene sequencing data at OTU level based on NMDS analysis (*P* calculated by PERMONAVA on the read count data) (error bars are SEM)

 Significantly higher protein groups from feed plants in the ileum (*P =* .0403), cecum (*P =* .0042), and colon (*P =* .0072) contents were observed in RYGB (Fig. 2a). This suggested a decrease in the capacity of the host to degrade plant-based feed. This was further reinforced by the fact that the plant protein groups exhibited higher label-free quantification (LFQ) values in RYGB hosts (*P* < .001 in all three sections) (Fig. 2b).

In the ileum, 40–50% of all protein groups were bacterial, while in the cecum and colon, this was approximately 80% (Fig. 2a). In RYGB, significantly lower relative numbers of bacterial protein groups were observed in the cecum (*P =* .0058) and colon contents (*P =* .0067) as compared to Sham-BWM.

### RYGB greatly alters the taxonomic community structure in the intestine

The microbial community structure of the ileum, cecum, and colon was analysed by 16S rRNA gene profiling and metaproteomics (see Additional file 1: Figures S3 and S4 for relative abundances of taxa). α-Diversity, based on 16S rRNA gene sequencing reads, revealed no significant differences in richness (based on the number of operational taxonomic units), in Shannon effective (combination of richness and evenness) or PD Faith index (total length of branches from community phylogenetic tree) in the ileum (Fig. 2c). For the cecum (*P* = .0007) and the colon contents (*P* = .0467), the richness decreased in RYGB as compared to Sham-BWM. In RYGB as compared to Sham-BWM, the Shannon effective also decreased in the cecum content (*P* = .0221) and in the colon mucus (*P* = .0063). We also observed a significant decrease in the PD Faith index for the cecum samples (*P* = .0006) in RYGB compared to Sham-BWM (Additional file 1: Figure S5). There was a significant shift in community taxonomic structure between RYGB and Sham-BWM from ileum to colon (Fig. 2d), as shown by NMDS similarity analysis. This revealed a greater separation between RYGB and Sham-BWM samples in the cecum (*P* = .005) and colon (content *P* = .008; mucus *P* = .008) than in the ileum (content *P* = .023; mucus *P* = .013), suggesting a stronger effect of RYGB on the community structure in the distal intestinal tract. A larger dissimilarity between Sham-BWM samples than between RYGB samples was observed.

**Fig. 3:**
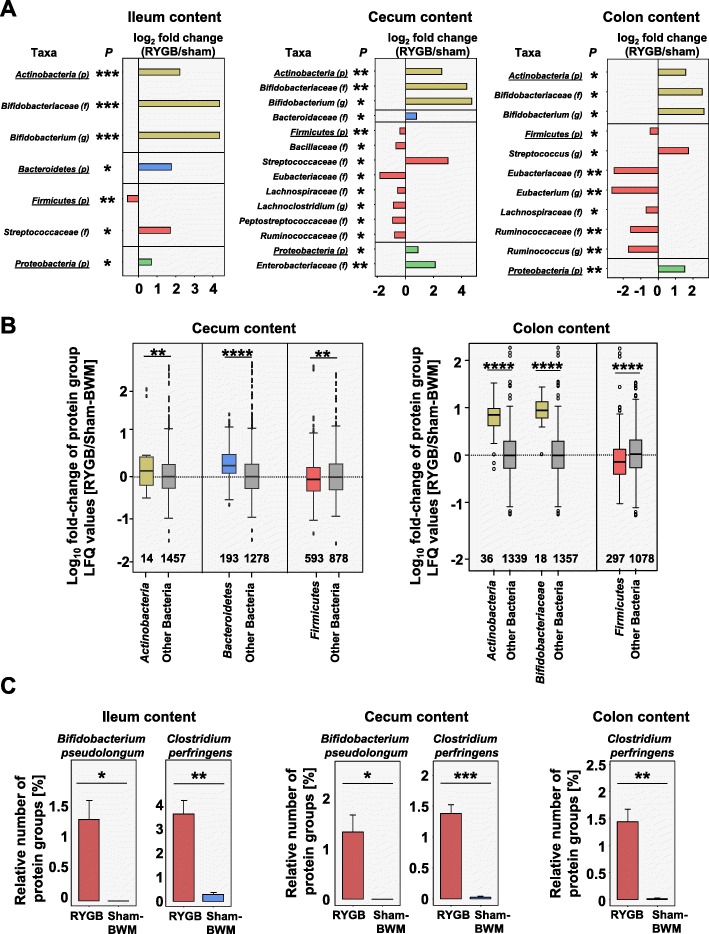
Community structure of active microbiota (*P* calculated by independent two-sided Student test and adjusted for multi-testing using the Benjamini-Hochberg method; ****P < .0001, ***P < .001, **P < .01, *P < .05). **a** Log_2_ fold change of relative numbers of protein groups based on all bacterial protein groups (p = phylum, f = family, and g = genus). **b** Boxplot depicting log_10_ fold changes of protein group intensities of those bacterial protein groups which were relatively quantifiable by label-free quantification (LFQ); numbers at the bottom of plot are the number of protein groups in boxplot. **c** Relative number of protein groups assigned to the species *B. pseudolongum* and *C. perfringens* (error bars are SEM)

Metaproteomics was used for taxonomic analysis, and as a result, metabolically active taxa of the microbiota should be highlighted [43]. The metaproteomic data was assessed on multiple taxonomic levels. On the phyla level, *Firmicutes* were observed as the most dominant phyla, with *Actinobacteria*, *Bacteroidetes*, and *Proteobacteria* making up substantial smaller fractions. The relative number of all *Firmicutes* protein groups was lower (ileum content *P* = .0021, cecum *P* = .0016, colon content *P* = .0082) for RYGB than for Sham-BWM (Fig. 3a). The lower LFQ values of quantifiable *Firmicutes* protein groups in RYGB for the cecum (*P* = .0039) and colon contents (*P* < .0001) (Fig. 3b) underlined this finding. A number of bacterial families from the *Firmicutes* were identified with fewer protein groups in RYGB as compared to Sham-BWM (Fig. 3a). Of these, *Eubacteriaceae* displayed the steepest decrease in the cecum (*P* = .0405) and colon contents (*P =* .0012), with the affiliated genus *Eubacterium* also showing a steep decrease in the colon content (*P* = .0019).

Interestingly, of all observed *Firmicutes* families, only *Streptococcaceae* in the ileum content (*P* = .0482) and cecum (*P* = .0406) and, at higher resolution, the affiliated genus *Streptococcus* in the colon content (*P* = .0370) were observed with increased relative number of protein groups in RYGB (Fig. 3a). This was in line with 16S rRNA gene sequencing data, where 18 operational taxonomic units (OTUs) of the cecum content and 6 from the colon content, assigned to *Streptococcus*, were relatively more abundant in RYGB as compared to Sham-BWM. These OTUs were mainly annotated to *Streptococcus hyointestinalis* (Additional file 1: Figures S8 and S10). Another interesting finding was that the *Firmicutes* species *Clostridium perfringens* was also observed at higher relative abundances in RYGB in the ileum content (*P* = .0056), cecum (*P* = .0007), and colon contents (*P* = .0097) on the metaproteome level (Fig. 3c). The 16S rRNA gene sequencing data underlined this finding by revealing *C. perfringens* was more abundant in RYGB than Sham-BWM (Additional file 1: Figures S6, S7, S8, S9, and S10).

The phylum *Actinobacteria* was more abundant in RYGB than in Sham-BWM (ileum content *P* = .0003, cecum *P* = 0.0016, colon content *P* = .0236), as indicated by the relative number of characteristic bacteria protein groups (Fig. 3a), and this is supported by the LFQ values of protein groups from *Actinobacteria* (cecum *P* = .0034, colon content *P* < .0001) (Fig. 3b). The *Actinobacteria* genus *Bifidobacterium* was significantly increased in RYGB as compared to Sham-BWM (ileum content *P* = .0002, cecum *P* = .0160, colon content *P* = .0370). Notably, the *Bifidobacterium* species *Bifidobacterium pseudolongum* showed significantly increased relative numbers of protein groups after RYGB in the ileum (*P* = .0412) and cecum contents (*P* = .0421) (Fig. 3c) in metaproteomic analysis. In the cecum content, all 5 OTUs annotated to *B. pseudolongum* were significantly more relatively abundant in RYGB than in Sham-BWM (Additional file 1: Figure S8).

Metaproteomics revealed that the phylum *Bacteroidetes* in the ileum content (*P* = .0104) and, at higher resolution, the affiliated family *Bacteroidaceae* in the cecum content (*P* = .0476) were more abundant in RYGB in comparison with Sham-BWM based on the relative number of protein groups and LFQ values (cecum *P* < .0001) (Fig. 3a, b). *Proteobacteria* were also observed at higher protein group abundances in the ileum contents (*P* = .0338), cecum contents (*P* = .0410), and colon contents (*P* = .0071).

In the mucus layer, the 16S rRNA gene sequencing data revealed shifts in taxonomic relative abundances on the OTU level between RYGB and Sham-BWM for the ileum and the colon (Additional file 1: Figure S7 and S9) with lower relative abundances of members of the genus *Lactobacillus* observed in RYGB.

### RYGB surgery modulates the functional structure of the microbiota

**Fig. 4:**
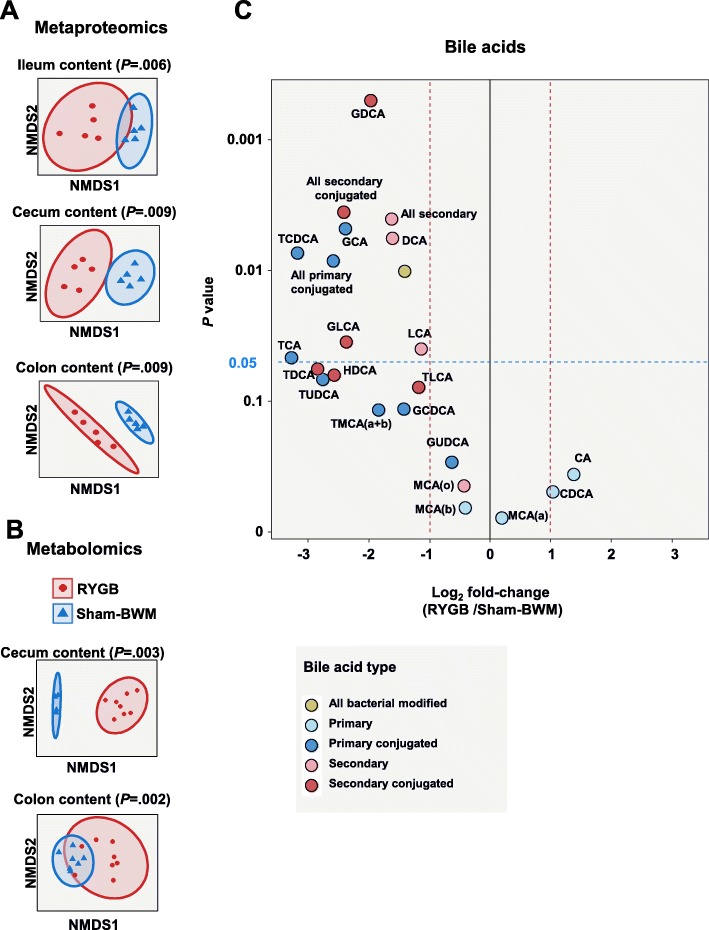
Functional structure of the microbiota. **a** Beta diversity of protein groups based on NMDS analysis (*n* = 5) (*P* calculated by PERMANOVA). **b** Beta diversity of metabolite concentrations from untargeted metabolomics based on NMDS analysis (cecum content RYGB *n* = 8 and sham *n* = 4; colon content RYGB *n* = 8 and sham *n* = 7) (*P* calculated by PERMANOVA). **c** Log_2_ fold change of bile acid concentrations detected in the colon content (RYGB *n* = 8 and sham *n* = 7). CA = cholic acid, CDCA = chenodeoxycholic acid, DCA = deoxycholic acid, GCA = glycocholic acid, GCDCA = glycochenodeoxycholic acid, GDCA = glycodeoxycholic acid, GLCA = glycolithocholic acid, GUDCA = glycoursodeoxycholic acid, HDCA = hyodeoxycholic acid, LCA = lithocholic acid, MCA(a) = alpha-muricholic acid, MCA(b) = beta-muricholic acid, MCA(c) = gamma-muricholic acid, TCA = taurocholic acid, TCDCA = taurochenodeoxycholic acid, TDCA = taurodeoxycholic acid, TLCA = taurolithocholic acid, TMCA(a + b) = tauromuricholic acid (alpha+beta), TUDCA = tauroursodeoxycholic acid (*P* have all been adjusted for multi-testing using the Benjamini-Hochberg method; ****P < .0001, ***P < .001, **P < .01, *P < .05)

To assess the functional consequences of bypass surgery on gut microbiota, the metaproteomics results were analysed with targeted and untargeted metabolomics data from the cecum and colon contents. NMDS similarity comparison of protein group intensities from the metaproteomic analysis and NMDS similarity comparison of MS-feature peak intensities from the untargeted metabolomics revealed significant global differences between RYGB and Sham-BWM (Fig. 4a, b).

### Targeted metabolomics reveals a shift in the metabolite profiles of the cecum and colon contents after RYGB

Many bile acid species in the colon were less abundant in RYGB than in Sham-BWM, while no single bile acid species was more abundant (Fig. 4c). Summed concentrations for primary conjugated (*P* = .0084), secondary (*P* = .0040), secondary conjugated (*P* = .0036), and all bacterial modified bile acids (*P* = .0101) were all lower in RYGB than in Sham-BWM (Fig. 4c). In the cecum, no difference in bile acid abundance was detectable between RYGB and Sham-BWM.

The majority of amino acids in cecum or colon contents—including tyrosine, phenylalanine, histidine, and branched-chain amino acids—were found at lower concentrations in RYGB than in Sham-BWM (Additional file 1: Figures S11 and S12). In contrast, the amines dopamine (*P* = .0439), L-3,4-dihydroxyphenylalanine (DOPA) (*P* = .0442), histamine (*P* = .0002), and spermine (*P* = .0180) were detected at higher concentrations in the RYGB colon content (Additional file 1: Figure S12C).

### RYGB decreases microbial arginine biosynthesis

**Fig. 5:**
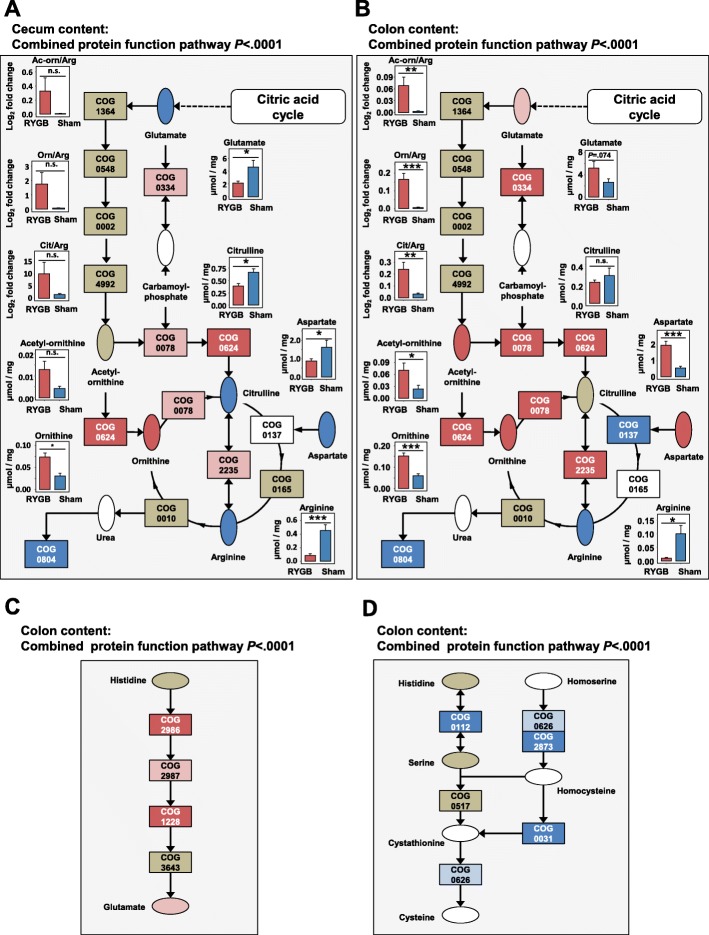
Functional changes in the intestinal microbiota after RYGB. For pathway maps: rectangles—protein functions with cluster of orthologous groups code; red—greater presence in RYGB; pink—tendency to greater presence in RYGB; dark blue—greater presence in sham; light blue—some evidence of greater presence in sham; khaki—protein function detected; white—protein function not detected. Selected metabolites ellipsis. Red—significantly higher concentrations in RYGB; pink—trend in higher concentration in RYGB; blue—significantly higher concentrations in sham; khaki—detected but non-significant. (****P < .0001, ***P < .001, **P < .01, *P < .05). **a** Whole microbiota metabolic pathway of arginine metabolism (modified KEGG 00220 map) in the cecum content. **b** Whole microbiota metabolic pathway of arginine metabolism (modified KEGG map 00220) in the colon content. **c** Whole microbiota metabolic pathway histidine metabolism (modified KEGG map 00340). **d** Whole microbiota metabolic pathway cysteine metabolism (modified KEGG map 00270) (error bars are SEM)

The arginine metabolic pathway was significantly altered in RYGB as compared to Sham-BWM, as observed in the cecum (*P* < .0001) and the colon contents (*P* < .0001) (Fig. 5a, b). In the cecum content, glutamate (*P* = .0113), citruline (*P* = .0117), aspartate (*P* = .0273), and arginine (*P* = .0001) were detected at lower concentrations in RYGB. Ornithine (*P* = .0178) was detected at higher concentrations in RYGB (Fig. 5a). This was in agreement with the metaproteomics results for this pathway. Here, protein groups were also observed at different abundances between RYGB and Sham-BWM—especially the acetylornithine deacetylase (COG0624) protein groups, of which all eight were only observed in RYGB (*P* = .004). Changes in the relative number of protein groups or their LFQ values from this pathway were stronger in the colon content. Here, ornithine carbamoyltransferase (COG0078; *P* = .0038) protein groups were detected in significantly higher relative numbers in RYGB than in sham. Of the eight protein groups detected only in one sample group, seven were detected only in RYGB. All four significantly regulated glutamate dehydrogenase (COG0334) protein groups had higher abundances in RYGB. In addition, another three glutamate dehydrogenases (COG0334) were only detected in RYGB.

In the colon content, all acetylornithine deacetylase (COG0624) protein groups which were only found in one sample group were only seen in RYGB. Five arginine deiminase (COG2235) protein groups were also only detected in RYGB. In contrast, all protein groups assigned to the function of the argininosuccinate synthase (COG0137) that were only identified in one sample group were only observed in Sham-BWM samples. This metaproteomic data agreed with the metabolomics data to a high extent with aspartate (*P* = .0001), acetyl-ornithine (*P* = .0354), and ornithine (*P* = .0014), all at a higher concentration in RYGB. There was a tendency towards increased concentrations of glutamate (*P* = .0745) in RYGB. Likewise, the ratios of acetyl-ornithine to arginine (*P* = .0075), ornithine to arginine (*P* = .0008), and citrulline to arginine (*P* = .0042) were significantly higher in RYGB (Fig. 5b). Arginine was detected at lower concentrations in RYGB (*P* = .0131).

### Histidine degradation and cysteine biosynthesis pathways were altered after RYGB

We observed changes in a number of other amino acid metabolic pathways. These included the histidine degradation pathway and cysteine biosynthesis pathway. For the histidine degradation pathway, protein groups were observed at higher relative numbers or were more abundant in RYGB (Fig. 5c). All seven unique protein groups for this pathway were only identified in RYGB. The relative number of protein groups of the function imidazolonepropionase (COG1228; *P* = .006) and histidine ammonia-lyase (COG2986; *P* = .024) were significantly higher in RYGB. Furthermore, cysteine biosynthesis pathway was significantly downregulated in RYGB (Fig. 5d). The relative abundances of both cysteine synthase (COG0031; *P* = .0044) and glycine/serine hydroxymethyltransferase (COG0112; *P* = .0246) protein groups were both significantly lower in RYGB. Also, all 16 cysteine synthases observed as unique were only identified in sham. Furthermore, of the 18 O-acetyl homoserine sulfhydrylase (COG2873) protein groups which were quantifiable, all were seen at lower abundances in RYGB of which 12 were significantly lower.

### Network analysis of omics data reveals highly modular functionality

**Fig. 6:**
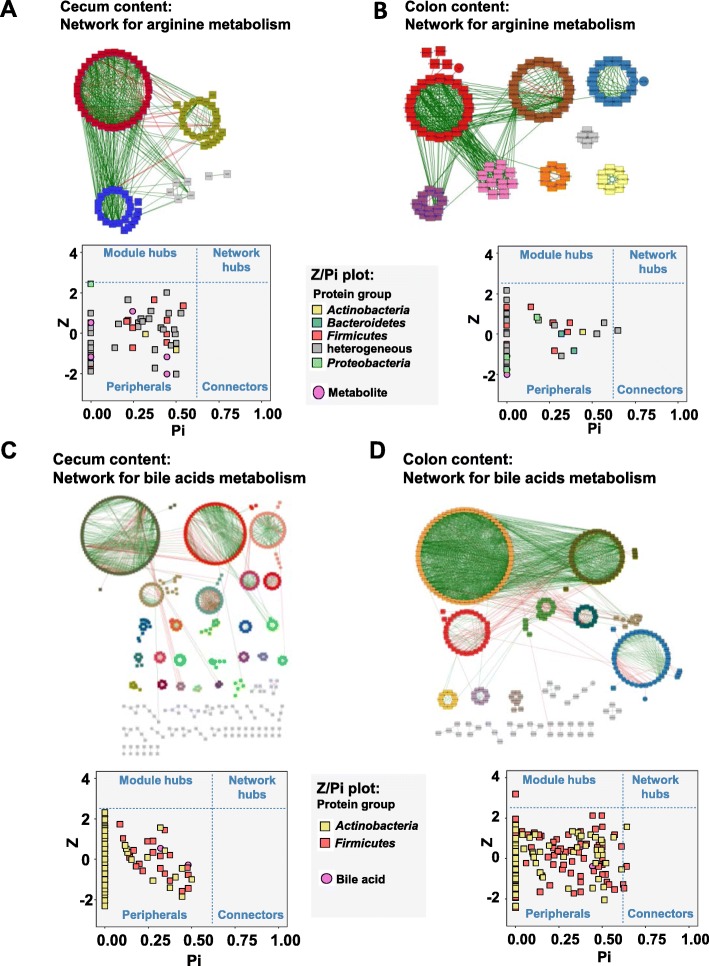
Network analysis of protein groups (squares) and metabolites (circles). Node colours in networks depict different clusters. Only nodes with *P* < .05 (Benjamini-Hochberg adjusted) shown. Analysis from the arginine pathway including scatterplot plotting within-module connectivity (Z) against among-module connectivity (Pi) for the cecum content (**a**) and colon content (**b**). Analysis of protein groups (squares) and bile acids (circles) including scatterplot plotting within-module connectivity (Z) against among-module connectivity (Pi) for the cecum content (**c**) and colon content (**d**)

Co-occurrence and co-excluding network analysis revealed highly complex interaction patterns between taxa, protein functions, and metabolites for RYGB (967 correlations with *P* < .01) and for Sham-BWM (1009 correlations with *P* < .01) (Additional file 1: Figure S13). Interestingly, the majority of correlations were between the different gut locations (400 in RYGB and 422 in sham) rather than within each location, which suggests strong upstream-downstream functional associations within the gut microbiota. In addition, a shift in the number of correlations within locations was observed from colon content to cecum (RYGB cecum 220 and colon content 141; Sham cecum 151 and colon content 254). Since significant changes in the arginine pathway and bile acid concentrations were observed, networks for RYGB were calculated to link relevant protein groups to arginine pathway metabolites and bile acids. These networks were calculated for cecum and colon content from RYGB data (Fig. 6a–d). For both the bile acids and arginine pathway metabolites, highly modular network patterns were observed with no or only very few connectors and modular hubs. No network hubs were observed. These findings suggest that taxa in the gut are highly specialised with regard to arginine metabolism and bile acid metabolism.

### Analysis of key drivers altering the microbiota after RYGB

**Fig. 7:**
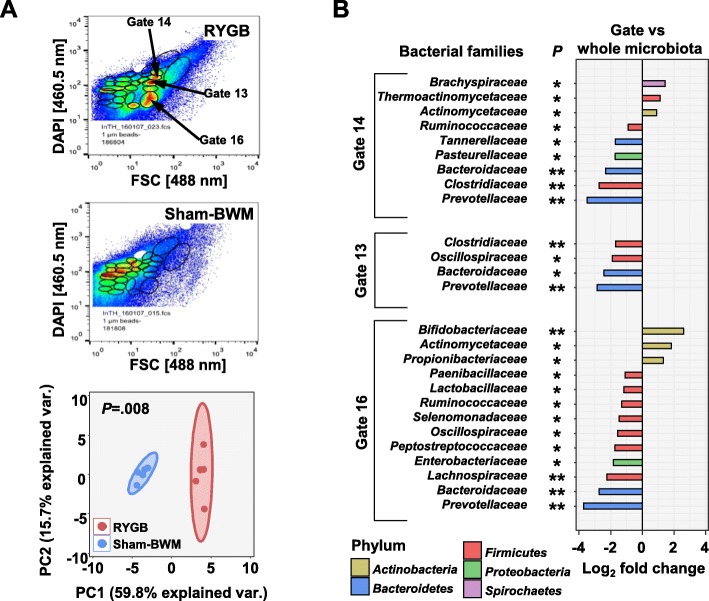
Comparison of sorted bacterial cells from RYGB cecum content with the entire microbiota from RYGB cecum content. **a** Flow cytometric analysis of cecum content samples. Staining by DAPI vs. FSC. Top: PCA analysis of cell counts in the 27 set gates from RYGB and sham samples (*n* = 5). Middle: representative pattern of RYGB samples. Bottom: representative pattern of sham samples. *P* calculated by PERMANOVA of gate bacterial cell counts (gates highlighted were those chosen for bacterial cell sorting). **b** Significant differences in bacterial family abundances based on relative number of protein groups between gate-sorted cecum samples with whole cecum samples

For determining the key driving force behind the changes in the microbiota, flow cytometric patterns of bacterial cells from cecum content were recorded (for representative patterns, see Fig. 7a, top and middle). The patterns were observed to be significantly different (*P* = .008) between RYGB and Sham-BWM (Fig. 7a, bottom). Three gates exhibiting higher bacterial cell counts for RYGB than for Sham-BWM (Fig. 7a, top and middle) were chosen for flow cytometric cell sorting of RYGB samples and collecting of bacterial cells for gate-specific metaproteomic analyses. The gate-specific metaproteomic data was compared to the non-sorted RYGB cecum metaproteome data to determine specific drivers in RYGB. Higher relative numbers of protein groups from the *Actinobacteria* family’s *Bifidobacteriaceae* (gate 16 *P* = .001), *Actinomycetaceae* (gate 13 *P* = .0436, gate 16 *P* = .0208), and the *Propionibacteriaceae* (gate 16 *P* = .0297) were observed for sorted bacterial cells as compared to the non-sorted cecum samples (Fig. 7b). Functional pathways for sorted bacteria cells involved in transcription, translation, ribosome functions, and folding of proteins were generally observed as enriched in protein groups as compared to the non-sorted bacteria, which suggests higher metabolic activity of these sorted bacteria as compared to the non-sorted bacteria (Additional file 1: Figure S14A, B, and C).

## Discussion

### Altered host plasma metabolites

Sphingomyelins are known to have a plethora of functions including as a building block of the cell membrane and as receptor molecules for a number of extracellular ligands [44]. In mice, lower levels of sphingomyelins in blood have been linked to an attenuation of atherogenesis [45], and a further study showed increased insulin sensitivity at lower sphingomyelin levels [46]. Of the 15 sphingomyelins we measured in the host plasma, concentrations of five were significantly altered in RYGB compared to Sham-BWM. All of them were found at lower concentrations in RYGB. Previous studies have observed a decrease in sphingomyelins after weight loss in humans [47] and after RYGB induced weight loss [48]. Interestingly, in our study, the control group was kept at the same body weight as the RYGB group thereby excluding an effect directly induced by weight loss. Our results therefore suggest a further influence of RYGB on the sphingomyelin levels of the host plasma.

### Community changes after RYGB show reduced diversity in a location-specific manner

Microbial diversity is strongly reduced after RYGB, which previous studies have also found, although studies performed on human samples could not allocate the changes in faeces to specific structures in the gut [49, 50]. Lower diversity is often an indicator of dysbiosis, a condition where the microbiome is disturbed leading to a decrease in microbial interaction within the community [12, 51]. Dysbiosis is linked to a number of detrimental health effects such as inflammatory bowel disease [12]. In our study, the intestinal environment was greatly altered by the RYGB surgery thereby disturbing the microbial community. The reduced diversity was detected 14 weeks after surgery and might change later due to the adaption of the microbiota to the altered intestinal environment. Studies in humans have found changes in diversity after bariatric surgery in a time-dependent manner [52].

At low taxonomic resolution, changes in composition were generally observed in all compartments of the gut. *Firmicutes* were observed at lower relative abundances in RYGB, as has previously been reported [52, 53]. As an exception, *Streptococcaceae* in the cecum and, at higher taxonomic resolution, *Streptococcus* in the colon was more relatively abundant in RYGB, which confirms previous studies [54]. In addition, a stronger presence of *Proteobacteria*, especially *Enterobacteriaceae*, observed after RYGB in the cecum and colon corresponds to observations in other studies [18, 53, 55]. Aron-Wisnewsky et al. [49] noted that these changes could not be linked to weight loss or the consequences of weight loss with certainty, but a comparison with other studies is hampered by the fact that most of them assess microbiota composition from 16S rRNA measurement from faeces, which does not necessarily reflect the composition in the ileum or cecum [30]. Here, taking advantage of a control group with matched body weights, our data suggests that the investigated parameters are related to the changed physical/chemical environment in the gut after anatomical rearrangement and not to weight loss. Recently, Liou et al. revealed in a mouse model that recipients of a microbiota transplant from RYGB-treated mice had decreased body weights without caloric restriction suggesting that the microbiota has a role in reducing adiposity after RYGB [19].

Other studies also observed the greater relative abundance of *Actinobacteria* and its genus *Bifidobacterium* in RYGB. Murphy et al. [56] identified an increase in *Actinobacteria* after RYGB. Flow cytometry combined with metaproteomics revealed the functional importance of *Actinobacteria* in RYGB, as these belonged to the more metabolic active part of the microbiota. Two studies of human stool samples reported a decrease in *Bifidobacterium* after RYGB [50, 52]. However, in the present study, the microbiota was investigated in lumen and mucus within the gut. The decrease in *Bifidobacterium* in human stools might be linked to weight loss after surgery and not to changed gut anatomy, a problem resolved in the present study by controls with matched body weights. In line with the presented data, low *Bifidobacterium* numbers associated with obesity were previously reported [57, 58]. In a test of symbiotic supplementation of fructose, *Lactobacillus*, and *Bifidobacteria* after bariatric surgery, no positive effects were found in terms of weight loss or inflammation [59].

### Functional changes in the microbiota are specific to different gut sections

A general increase in amines, such as dopamine and its precursor DOPA, and histamine and spermine, was observed in the colon of RYGB. The elevated concentrations of these metabolites may be caused by the higher relative abundance of *Proteobacteria* and *Streptococci*, which are known to synthesise these amines by decarboxylation of the corresponding precursor amino acids [53, 60]. It has been suggested that this strategy is employed by bacteria for microbial acid resistance [61]. The importance of these amines as bioactive or toxic gut components remains largely unaddressed and is an ongoing matter of discussion [62] although many are known to be involved in host signalling pathways [63].

The functional interactions of microorganisms have a profound role in human health and disease [64]. These interspecies interactions can have beneficial, neutral, or harmful effects on the microbiota. The network analyses revealed numerous interspecies and inter-gut section interactions, which suggest high dynamic community composition and assembly. The RYGB surgery perturbed the established functional community interactions which were observed in the colon content and, to a lesser extent, in the cecum content. In the arginine metabolism, large increases in the relative number and abundances of protein groups and metabolites were observed, while arginine itself was present at lower levels in RYGB. These changes can be explained by the combination of the regulation of the pathway since lower levels of arginine induce a higher abundance of proteins involved in synthesis and, on the other hand, by feedback inhibition of the glutamate dehydrogenase by arginine [65]. In addition, due to the decrease in arginine, an increase in the concentration of aspartate in the colon was observed. Aspartate is one of the main sources of microbial-derived propionate [66]. Previous studies identified that diets supplemented with arginine could restrict *C. perfringens* growth in broiler chickens [67]; this may also explain why *C. perfringens* was seen at higher levels in our study since arginine was observed at lower concentrations in RYGB.

Histidine degradation is tightly controlled in bacteria because of the high energy costs required in its synthesis [68]. We observed an increase in the abundance of protein groups involved in the degradation of histidine to glutamate in the colon of RYGB compared to Sham-BWM. Interestingly, the decarboxylation degradation product of histidine namely histamine is increased in the colon of RYGB. Histamine is a known signalling molecule for the immune system [69]. A number of bacteria species, especially from the *Proteobacteria*, are able to synthesise histamine in the gut. We observed an increase in *Proteobacteria* which could explain the increase in the levels of histamine in the colon of RYGB.

Cysteine synthesis is known to occur in the intestinal microbiota [70]. Cysteine is a precursor of bacterial produced hydrogen sulphide in the gut. At lower levels, hydrogen sulphide has beneficial health effect but at excessive higher concentrations can contribute to colonic pathology [70]. Protein groups from the microbiota involved in cysteine synthesis were observed at lower abundances in the colon of RYGB compared to Sham-BWM. The metaproteomic data suggest a decrease in the cysteine synthesis, and therefore, cysteine concentration should be lower in the colon of RYGB that may have an impact on the health of the host.

Recent studies have shown that the exchange of amino acids greatly contributes to the interactions and composition of microbial communities [64]. Mee et al. highlighted that more than 98% of microbial genomes lack essential pathways or key genes for the synthesis of amino acids [71]. Thus, most microorganisms are auxotrophic and require extracellular sources of amino acids. We observed that the majority of amino acids in cecum or colon contents, including tyrosine, phenylalanine, histidine, and branched-chain amino acids, were found at lower concentrations in RYGB than in Sham-BWM (Additional file 1: Figures S10 and S11). Aromatic amino acids, such as phenylalanine, tyrosine, and histidine, are energetically more costly to synthesise than simpler amino acids [72]. The metabolic costs for the synthesis of amino acids do vary and depend on which metabolic pathways are present in the microbe [72]. Since RYGB should change the nutrient composition in the gut, the original auxotrophic interactions, growth behaviour, and taxonomic structure should be altered.

### Alteration in bile acid profile may drive changes in the distribution of microbiota

Apart from nutrients altering the microbiota, bactericidal agents such as bile acids may also greatly influence the microbiota composition by disrupting bacterial cell membranes [73–75]. Thus, the observed changes in bile acid concentrations in RYGB were an important finding. Previous studies have shown alterations in blood bile acid concentrations after gastric bypass [76] and after bile diversion [77]. Host-derived conjugated bile acids are deconjugated in the gut by specific microbes, thus enhancing their toxicity [78]. The lower levels of all primary and secondary conjugated bile acids in the colon content are consistent with the observed higher relative abundances of *Bifidobacterium*, *Lactobacilli*, and *C. perfringens*. These are known to deconjugate bile acids [79–81]. Bacterial dehydroxylation of bile acids to the secondary bile acid increases hydrophobicity and thus strengthens toxicity [82]. Numerous taxa associated with dehydroxylation of bile acids such as *Firmicutes* (*Clostridium* or *Eubacterium*) [81] were less abundant in the RYGB colon. We could not observe alterations in the bile acid profile in the cecum, probably because this process depends on time and transport. These low concentrations of bile acids might be an important factor that induces changes in the microbiota structure in RYGB. *Bacteroidetes* and *Actinobacteria*, especially *Bifidobacteria*, are known to be more susceptible to bile acids than *Firmicutes* [78] which may explain our findings that the former were generally present at higher relative abundances in RYGB. Furthermore, in the colon content, most OTUs assigned to *Allobaculum* were only present in Sham-BWM. This finding is consistent with other studies where *Allobaculum* were detected at higher relative abundances when higher concentrations of bile acids were present [78]. *C. perfringens* and *Lactobacilli* are also inhibited by DCA [78, 83]. *C. perfringens* and *Streptococci*, which are members of the *Lactobacilli*, appear at higher relative abundances in RYGB and therefore support our assumption that bile acid concentrations are an important factor in shaping the intestinal microbiota. Changes in bile acid profiles are reported to affect the metabolism of the host via the farnesoid X receptor [84], which also controls glucose and lipid metabolism in the liver as well as bile acid synthesis. A recent study in mice revealed that treatment with the antioxidant tempol indirectly inhibited the FXR signalling in the gut [85]. The inhibition was conveyed by tempol altering the microbiome which led to a decreased bile acid hydrolase activity in the community and a resulting increase in the bile acid tauro-b-muriccholic acid, an antagonist for the farnesoid X receptor [85].

## Conclusions

Our study revealed that RYGB independent of weight loss remarkably changed the taxonomic structure of the microbiome and more importantly the actual functionality in the microbial community. Functional changes in the microbiota are specific to different gut sections. Our findings support the hypothesis that alteration in bile acid profile may drive changes in the distribution of microbiota. Bile acids are key players in shaping community composition and host metabolism. In addition, abundance changes in the amino acid and amines have a great impact in regard to host health mediated by microbiota. Amines and their precursor’s amino acids play an important role as signalling molecules for a number of host processes. However, complex interplay between toxicity and metabolism by specific bacteria requires further investigation as well as the interdependence of the microbial and the host metabolisms influenced by bile acids and other metabolites. An in-depth understanding of these relationships has the potential to facilitate the design of probiotic approaches that can be used to supplement bariatric surgery in the future.

## Supplementary information

**Additional file 1.** Supplement includes detailed methods, supplemental figures, results and discussion.

**Additional file 2.** Omics data from 16S rRNA gene sequencing, metaproteomics and targeted metabolomics.

## Data Availability

Data is available in the additional files. The mass spectrometry proteomics data have been deposited to the ProteomeXchange Consortium via the PRIDE [86] partner repository with the dataset identifier PXD013337. The 16S rRNA gene sequencing data has been deposited in SRA database (https://www.ncbi.nlm.nih.gov/sra) under the accession number PRJNA561349.
