# Supplementary material for: Gastric bypass surgery in a rat model alters the community structure and functional composition of the intestinal microbiota independently of weight loss
Source: Microbiome. 2020 Feb 7;8:13. doi: 10.1186/s40168-020-0788-1 (PMC7007695; doi:10.1186/s40168-020-0788-1)
Supplement: Supplementary file 2 — Additional file 1. Supplement includes detailed methods, supplemental figures, results and discussion. [file 40168_2020_788_MOESM1_ESM.docx]

**ADDITIONAL FILE 1: SUPPLEMENT**

## Gastric bypass surgery in a rat model alters the community structure and the functional composition of the intestinal microbiota independently of weight loss

Sven-Bastiaan Haange ^1, 2^, Nico Jehmlich ^1^, Ute Krügel ^3^, Constantin Hintschich ^4^, Dorothee Wehrmann ^1^, Mohammed Hankir ^4§^, Florian Seyfried ^5^, Jean Froment ^1^,Thomas Hübschmann ^6^, Susann Müller ^6^, Dirk K. Wissenbach ^1#^, Kang Kang ^7^, Christian Buettner ^8^, Gianni Panagiotou ^7, 9, 10^, Matthias Noll ^8^, Ulrike Rolle-Kampczyk ^1^, Wiebke Fenske ^4^, Martin von Bergen ^1,2^

^1^ Department of Molecular Systems Biology, Helmholtz Centre for Environmental Research-UFZ, Leipzig, Germany

^2^ Institute of Biochemistry, Faculty of Life Sciences, University of Leipzig, Leipzig, Germany

^3^ Rudolf Boehm Institute of Pharmacology and Toxicology, Medical Faculty, University of Leipzig, Leipzig, Germany

^4^ Neuroendocrine Regulation of Energy Homeostasis Group, IFB Adiposity Diseases, Leipzig, Germany

^5^ Department of General, Visceral, Vascular and Pediatric Surgery, University of Würzburg, Germany

^6^ Department of Environmental Microbiology, Helmholtz Centre for Environmental Research-UFZ, Leipzig, Germany

^7^ Leibniz Institute for Natural Product Research and Infection Biology, Hans Knoll Institute, Jena, Germany

^8^ Institute for Bioanalysis, Faculty of Applied Sciences, Coburg University of Applied Sciences and Arts, Coburg, Germany

^9^ Systems Biology & Bioinformatics Group, School of Biological Sciences, Faculty of Science, The University of Hong Kong, Hong Kong S.A.R., China

^10^ Department of Microbiology, Li Ka Shing Faculty of Medicine, The University of Hong Kong, Hong Kong S.A.R., China

§ Current address: Department of Experimental Surgery, Wuerzburg University Hospital, Wuerzburg, Germany

# Current address: Institute of Forensic Medicine, Jena University Hospital, Jena, Germany

Table of Contents

[Supplemental Methods - 4 -](#_Toc27483109)

[Abdominal surgeries and postoperative care - 4 -](#_Toc27483110)

[RYGB procedure - 4 -](#_Toc27483111)

[Sham surgery - 4 -](#_Toc27483112)

[Flow cytometry and cell sorting of microbial community samples - 5 -](#_Toc27483113)

[Bacteria cell lysis for DNA and protein extraction - 7 -](#_Toc27483114)

[DNA purification - 8 -](#_Toc27483115)

[16S rRNA gene sequencing - 8 -](#_Toc27483116)

[Protein in-gel digestion - 9 -](#_Toc27483117)

[LC-MS/MS measurements - 11 -](#_Toc27483118)

[Metaproteomics: bioinformatics, statistics and data analysis - 13 -](#_Toc27483119)

[Metabolite extraction from samples - 15 -](#_Toc27483120)

[Targeted metabolomics - 15 -](#_Toc27483121)

[Untargeted metabolomics - 17 -](#_Toc27483122)

[Network analysis - 18 -](#_Toc27483123)

[Supplemental Tables and Figures - 21 -](#_Toc27483124)

[Supplemental Results - 37 -](#_Toc27483125)

[Changes in animal body weight and host phenotypical parameters - 37 -](#_Toc27483126)

[Sequencing data - 37 -](#_Toc27483127)

[Metaproteomics - 38 -](#_Toc27483128)

[Changes in Taxonomic distribution after RYGB - 38 -](#_Toc27483129)

[Protein groups involved in iron utilisation in the ileum after RYGB - 42 -](#_Toc27483130)

[Bacterial xylan degradation and utilisation metabolic pathways are more active in RYGB cecum content - 43 -](#_Toc27483131)

[Analysis of metabolic pathways of cell sorted bacteria - 44 -](#_Toc27483132)

[Functional and taxonomic description of the microbiota detected in the biliopancreatic limb lumen contents - 44 -](#_Toc27483133)

[Supplemental Discussion - 46 -](#_Toc27483134)

[Functional changes in the microbiota are more localised in the gut - 46 -](#_Toc27483135)

[Xylan metabolism altered in the microbiota - 47 -](#_Toc27483136)

[Species found at high abundance in the gut after RYGB are probably seeded from the microbiota observed in the biliopancreatic limb - 47 -](#_Toc27483137)

[Supplemental References - 49 -](#_Toc27483138)

## Supplemental Methods

### Abdominal surgeries and postoperative care

All abdominal surgical procedures were done after an overnight fast. The RYGB procedure was performed by a bariatric surgeon according to a previously established protocol [[1](#_ENREF_1), [2](#_ENREF_2)]. Briefly, animals were anaesthetized with 5% isoflurane in 2 L/min oxygen and maintained on 2-3% isoflurane in 0.5 L/min oxygen. Following induction of anaesthesia, the abdominal wall was opened by a midline incision.

### RYGB procedure

The jejunum was transected 15 cm aboral to the pylorus to create a 10 cm biliopancreatic limb. The proximal end was anastomosed to the ileum approximately 25 cm oral from the cecum, creating the common channel. The stomach was then transected 3 mm aboral to the gastroesophageal junction. At the proximal end, the gastric pouch (2-3% original stomach size) was anastomosed in an end-to-side fashion to the distal end of the small bowel forming the alimentary limb. At the distal end, the gastric remnant was closed using continues sutures.

### Sham surgery

The small bowel and the gastroesophageal junction were mobilised, and a 1 cm long gastrostomy was performed on the anterior wall of the stomach with subsequent closure.

For postoperative analgesia, carprofen (5 mg/kg i.p.) was administered intraoperatively and on postoperative days 1 and 2. During the post-operative recovery period (7 days) animals were monitored for signs of pain, distress or morbidity every 12 hours.

### Flow cytometry and cell sorting of microbial community samples

##### Fixating and staining of Bacteria cells from cecal content samples

Cecal content samples of RYGB (n=5) and Sham (n=5) were suspended, vortexed, and washed with phosphate buffered saline once (PBS, 6 mM Na2HPO4, 1.8 mM NaH2PO4, 145 mM NaCl, pH 7, 3,200 g, 10 min, 4 °C) and the bacterial cells were stabilized by adding 2 ml paraformaldehyde solution (PFA, 2% in PBS) to the cell pellet and incubated for 30 min at room temperature (RT). After another centrifugation step (3,200 g, 10 min, 4 °C), 4 mL of ethanol (70%) were added for fixation and the cell solution stored at -20 °C in 1 mL PBS (1.8 g/L Na_2_HPO_4_, 0.223 g/L NaH_2_PO_4_, 8.5 g/L NaCl, pH 7.2).

One hundred µl of the fixed samples were vortexed, washed with PBS for 20 min, centrifuged at 3,200 g, 10 min, 4 °C and the resulting cell solutions were each adjusted to an OD of 0.035 (dʎ700nm = 5 mm) with PBS. 2 mL of an adjusted sample were centrifuged (3,200 g, 10 min, 4 °C), and the pellet resuspended with 1 mL solution A (0.11 M citric acid and 4.1 mM Tween 20, with distilled water) and incubated at RT for 10 min in an ultrasonication bath (Merck Eurolab, Darmstadt, Germany) and 10 min without any further treatment. After another centrifugation step (3,200 g, 10 min, 4 °C) the cells were stained with 3.5 ml solution B [0.5 μM DAPI (4',6-diamidino-2-phenylindole) in phosphate buffer (289 mM Na2HPO4 and 128 mM NaH2PO4 in distilled water)] at RT and stored over night at 4°C.

*Flow cytometry and data analysis*

Fixed and DNA-stained bacteria cells were measured with an Influx for the measurement: BD Influx v7 Sorter USB applying the BD FACS Software 1.2.0.142 (Becton, Dickinson and Company, Franklin Lakes, NJ, USA). The instrument was equipped with a 488 nm Sapphire OPS laser (400 mW) and a 355 nm Genesis CX laser (100 mW, both Coherent, Santa Clara, CA, USA). The 488 nm laser light was used for the detection of the forward scatter (FSC, 488/10) and side scatter (SSC, 488/10, trigger signal).250,000 bacterial cells per sample were measured by recording relative cell size (forward scatter [FSC]) and chromosome numbers per cell (DAPI staining). The DAPI fluorescence was measured at PMT9 (460/50) after excitation with 355 nm laser light.

The fluidic system was run at 33 psi using a 70 μm nozzle. The sheath fluid consisted of 0.5 x FACSFlow buffer (BD). For optical alignment of the cytometer we used the following beads: 1 μm blue fluorescent FluoSpheres (Molecular Probes, F-8815, Eugene, OR, USA) and 2 μm yellow-green fluorescent FluoSpheres (ThermoFisher Scientific, F8827, Waltham, MA, USA)

For comparison of samples, we spiked 0.5 μm and 1 µm UV Fluoresbrite Microspheres (Polysciences, 18339, Warrington, PA, USA) in each of the samples. The microspheres served as internal standards. Cell data were collected in logarithmically scaled 2D-dot plots according to DAPI fluorescence for DNA content and forward scatter (FSC) for cell size-related information. Samples were sorted with 20.000 events/sec, 4way-sort, 1drop-pure, drop frequency 69.8 kHz, drop delay: 34.6063. A principal component analysis was performed on the 2Dplotpatterns from all samples. Significance between 2Dplotpatterns recorded for RYGB and sham was calculated by PERMANOVA on the gate cell counts using the Adonis function in the R-package vegan.

*Bacteria cell sorting*

The flow cytometry patterns of RYGB and sham samples from the cecum content were compared. Three gates (Gates 13, 14 and 16) were chosen based on high bacterial cell counts detected in RYGB samples and low counts in sham samples. From each gate 5*10^6^ bacterial cells (n=4) were collected based on a modified previously described method [[3](#_ENREF_3)]. Briefly, the sorted cells were then transferred on to 0.22 hydrophilic low protein binding membrane micro titer plate (MultiScreen HTS^TM^ GV Sterile Plate, Millipore). This plate was first vacuum treated at 12 inHg vacuum, the membrane was washed with 2 times 300 µl bidest, then the sorted cells were positioned within 300 µl bidest on the membrane, afterwards the cells were washed 3 times with 300 µl bidest, respectively and the plate was kept under vacuum for another 20 min. Following the plastic material was removed from the downside of the micro titer plate using a scalpel, then the membrane was cut out and sored under sterile condition in an Eppendorf tube at -20 degree until further down processing.

These sorted bacteria samples were forwarded to protein extraction and metaproteomic analysis (see below).

### Bacteria cell lysis for DNA and protein extraction

Five replicates samples from the biliopancreatic limb lumen content, ileum mucus, ileum lumen content, cecum lumen content, colon mucus, colon lumen content each from RYGB rats and sham rats were randomly chosen for bacteria lysis. For content samples, approximately 0.5 g of sample was chosen, while the entire content of the mucus samples was used. These samples were thawed and resuspended in 1mL Lysis-buffer (50 mM Tris, 5 mM EDTA, 0.4% SDS, 50 mM NaCl, 1 mM PMSF, pH=8) and 4 glass beads as well as approx. 0.5g Zirconia beads were added to each sample. Three cycles of disruption by bead beating using a Fastprep (FastPrep -24, MP Biomedicals) was performed with the parameters set to 5.5 ms, duration 1min, 4°C. Between cycles, samples were kept on ice for 1 min. Then samples were heated in a Thermomixer (Thermomixer comfort 5355, Eppendorf) at 60°C with shaking at 1400 rpm for 15 min. This was followed by sonication using an ultrasound probe (UP50H, Hielscher) set with parameters cycle 0.5 and amplitude 60%. Sonication was done for one minute, followed by a minute rest on ice and sonication for a further minute. Samples were spun at 10,000 rcf at 4°C for 10 min. Supernatants, containing the DNA and protein content, were kept. The pellets were resuspended in 300 µL of Lysis buffer, and cell lysis was repeated. The resulting supernatant of each sample was added to the corresponding supernatant of the first bacteria lysis round and frozen at -20°C for storage.

### DNA purification

To all the supernatants from bacteria lysis of the mucus samples and half of the supernatant from the content samples 260µL 10M ammonium acetate was added, mixed and incubated on ice for 5 min. Then samples were spun at 20,000 rcf for 10 min at 4°C and the supernatants kept. Isopropanol in equal volume was added to the supernatants, mixed thoroughly and kept on ice for 30 min, and then centrifuged at 20,000 rcf for 15 min at 4°C to pellet DNA. The pelleted DNA was washed with 10 µL pure ethanol, dried in a speed vac and finally dissolved overnight at 4°C in TE-Buffer (1mM EDTA, 10mM Tris, pH=8). The dissolved DNA was purified and proteins removed using the QIAmp DNA Mini Kit (QIAGEN, USA) and following the manufacturer’s instructions. Finally, the DNA content recovered from each sample was quantified using Nanodrop (NanoDrop2000, Thermo Scientific). A negative control was also handled containing only the lysis buffer and subjected to the DNA purification steps.

### 16S rRNA gene sequencing

Free DNA was amplified by PCR using the specific 16S rRNA gene V4 region primer pair 515F/806R. These primers had a barcode region on the forward primer specific to the sample. PCR was performed using the HotStarTaq Plus Master Mix Kit (Qiagen, USA) under the following conditions: 94°C for 3 minutes, followed by 28 cycles of 94°C for 30 seconds, 53°C for 40 seconds and 72°C for 1 minute, after which a final elongation step at 72°C for 5 minutes was performed. In total 30 PCR cycles (5 cycles used on PCR products were done. PCR products from all samples were pooled in equal proportions based on their molecular weight and DNA concentrations followed by purification using calibrated Ampure XP beads. A DNA library from the pooled products was prepared by following the Illumina TruSeq DNA library preparation protocol. Sequencing was done on a Miseq following the manufacturer instructions at MR DNA (www.mrdnalab.com, Shallowater, TX, USA). Furthermore, MR DNA performed data analysis using their analysis pipeline. Briefly, using the QIIME [[4](#_ENREF_4)] to assess quality sequences were joined, depleted of barcodes then sequences <150bp removed, sequences with ambiguous base calls removed, sequences were denoised operational taxonomic units (OTU’S) generated and chimaeras removed as previously described [[5](#_ENREF_5), [6](#_ENREF_6), [7](#_ENREF_7), [8](#_ENREF_8), [9](#_ENREF_9), [10](#_ENREF_10)].OTU’s were generated by clustering sequence reads to bins with 97% sequence similarity [[11](#_ENREF_11)]. OTU’s were classified to taxa by blasting them against a curated database derived from GreenGenes, RDPII [[12](#_ENREF_12), [13](#_ENREF_13)] and NCBI (http://www.ncbi.nlm.nih.gov/). OTU’S were classified to as previously decribed [[14](#_ENREF_14)]. Briefly, OTUs were assigned to species if blast matches had similarities of > 97% OTU’s for matches with similarities of between 97% and 95% were annotated to unclassified genus. Matches of between 95% and 90% were annotated to unclassified family, of between 90% and 85% annotated to unclassified order, of between 85% and 80% annotated to unclassified class, of between 80% and 77% annotated to unclassified phylum and of < 77% annotated to unknown. Finally, the relative number of reads assigned to each of the bacteria taxa in each sample were used for taxonomic analysis. Statistical analysis of biologically relevant data and data visualisation was done using R. For OTU-level analysis the R package Rhea was used to normalise data, calculate alpha diversity and do statistical analysis [[15](#_ENREF_15)]. All p-values were corrected for multi-testing with the Benjamini-Hochberg method. The 16S rRNA gene sequencing raw fastq data has been deposited in SRA data base (<https://www.ncbi.nlm.nih.gov/sra>) under the accession number PRJNA561349

### Protein in-gel digestion

Only content samples were metaproteomic analysed (n=5, except the biliopancreatic samples and cecum cell-sorted samples n=4). Volumes of sample supernatants containing 150 µg of protein content, as determined with the BCA protein assay kit (Pierce Protein Biology Products, Thermo Fisher Scientific Inc., Rockford, USA); were precipitated overnight at -20°C with a 5-fold volume of acetone. The exception was the cell sorted cecum samples were all protein extract was precipitated. Before adding the acetone, the total volume of precipitation was normalised by adding lysis buffer to supernatant samples to equalise volumes. Next day samples were spun at 10,000 rcf at 4°C for 30min. Pellets containing the precipitated proteins were left to dry, resuspended in SDS-sample buffer (2% SDS, 2 mM beta-mercaptoethanol, 4% glycerol, 40 mM Tris-HCl pH 6.8, 0.01% bromophenol blue) and then were heated in a Thermomixer to 60°C for 10 min while shaking. Proteins were separated by 1-D SDS-PAGE using a 12% acrylamide separating gel and the Laemmli-buffer system [[16](#_ENREF_16)]. Samples were run for approximately 1.5 cm into the separation gel. To visualise proteins SDS-PAGE gels were stained overnight using a 50 mL colloidal Coomassie Brilliant blue G-250solution (Roth, Kassel, Germany)) and then were washed in ultrapure water for 3 h. Except for the cell sorted samples, each lane, containing the protein content from one sample, was cut into 5 equal sized pieces. These fractions were subsequently handled separately. For the cell sorted samples the entire lane was cut out as one fraction. Each fraction was cut into small pieces to increase the surface area of the gel pieces and then destained with 200 µL of 40% acetonitrile in10mM ammonium bicarbonate for 15 min while shaking. The solution was removed, and the destaining was repeated. Following this, the fractions were dried first chemically in acetonitrile for 5 min and then physically in a speed vac (Univapo 100H, UniEquip, Martinsried, Germany) at 40°C for 5 min. Cysteine residues were alkylated by first reducing disulphide bridges with 30 µL 10 mM 1.4-Dithioerythritol in 10 mM ammonium bicarbonate solution and shaking for 30 min, followed by alkylation with 30 µL of 2-Iodacetamide in 10 mM ammonium bicarbonate solution and shaking for 30 min. Fractions were chemically and physically dried, as previously described, washed for 10min with 200 µL 10 mM ammonium bicarbonate solution while shaking, and chemically and physically dried again. In-gel protein digestion was performed by adding 20 µL Trypsin solution (5 µg/mL; in 5% acetonitrile in 10 mM ammonium bicarbonate) to each fraction and leaving at 37°C overnight. Next day tryptic peptides were eluted from gel pieces by first adding 50 µL 5 mM ammonium bicarbonate, sonicating for 1 min in an ultrasound bath and followed by shaking for 10 min. For each fraction, the solution containing the eluted peptides was removed and stored. Then 60 µL extraction buffer (10% formic acid 50% acetonitrile) was added to gel pieces of each fraction and shaken for 10 min. The extraction buffer containing eluted peptides was removed and added to the corresponding peptide containing solutions from the previously eluting step. This was repeated, but using 70 µL extraction buffer. The samples containing the eluted tryptic peptides were dried in a speed vac. Samples were reconstituted in 15 µL 0.1% formic acid and desalted by solid phase extraction using ZipTip C18 (Merck Millipore, Germany) system and following the manufactures instructions, with a maximum of 25 µg of peptides per fraction desalted. The desalted peptides were dried in a speed vac and stored at -20°C.

### LC-MS/MS measurements

For the ileum and colon lumen content fractions, peptides were reconstituted in 25 µL 0.1% formic acid of which 7 µL for each fraction was injected for separation into a nano-HPLC system (UltiMate 3000, Dionex/Thermo Fisher Scientific, Idstein, Germany) coupled online with an Orbitrap Fusion mass spectrometer (Thermo Fisher Scientific, San Jose, CA, USA). Peptides were first trapped on a C18-reverse phase trapping column (Acclaim PepMap ® 100, 75 µm x 2 cm, particle size 3 µM, nanoViper, Thermo Fisher) and washed for 3 min with 0.1% formic acid with a 5 µL/min flow. The peptides were eluted using a 114 min linear gradient from 2% B to 55% B (A: 0.1% formic acid in MS-grade water; B: 80% acetonitrile, 0.1% formic acid in MS-grade water) followed by gradient 2 min gradient to 90% B, with 2 min isocratic flow, then 1 min gradient to 2% B and finally an isocratic flow for 10 min at 2% B. During the entire analytical run after trapping the solvent rate was set to 300 nL/min and the eluting peptides were separated on a downstream analytical column (Acclaim PepMap ® 100, 75 µm x 25 cm, particle size 3 µM, nanoViper, Thermo Fisher) kept at 35°C before being ionized by an Advion Triversa Nanomate ion source and entering the mass spectrometer. Ionized peptides were measured in a data dependent manner using the cycle time of 3 s, with the parameters set on the Orbitrap fusion for MS1: Detector type =Orbitrap, Resolution =120,000 Hz, Scan range =400-1600 m/z, Maximum injection time =80 ms, AGC-target =400,000 ions, S-lens RF Level =60, polarity =positive, data type =profile. The parameters for decision on precursors fragmentation were: set to the following: Filter Type =MIPS, Include charge state(s) =2-7, Filter DynamicExclusion, Exclude after n times =1, If occurs within (s) =30, Exclusion duration (s) =60, Mass tolerance =10 ppm, S/N threshold =2, Filter Type =IntensityThreshold, Signal Intensity =50,000 ions, Precursor Priority =MostIntense, MSn Level =2, Isolation Mode =Quadrupole, Isolation Window =1.6 m/z, , FirstMass =100 m/z, ActivationType =HCD, Collision Energy =28%. Parameters for MS2 measurements were: Detector Type =Orbitrap, Orbitrap Resolution =15,000 Hz, Scan Range =350-1400 m/z, Maximum Injection Time =120 ms, AGC Target =50,000 ions, DataType =Centroid, Polarity =Positive.

For the biliopancreatic limb and cecum lumen content fractions as well as the flow cytometry sorted cecum samples peptides were reconstituted in 25 µL 0.1% formic acid of which 7 µL for each fraction was injected for separation into nano-HPLC system (UltiMate 3000, Dionex/Thermo Fisher Scientific, Idstein, Germany) coupled online for analysis with an Orbitrap Q Exactive HF mass spectrometer (Thermo Fisher Scientific, San Jose, CA, USA). Peptides were first trapped on a C18-reverse phase trapping column (Acclaim PepMap ® 100, 75 µm x 2 cm, particle size 3 µM, nanoViper, Thermo Fisher) and washed for 5 min with 0.1% formic acid with a 5 µL/min solvent flow. Then peptides were eluted using a 90 min linear gradient from 4% B to 25% B (A: 0.1% formic acid in MS-grade water; B: 80% acetonitrile, 0.1% formic acid in MS-grade water) followed by a 30 min gradient from 25% B to 50% B and a 3 min gradient to 99% B with 5 min isocratic flow, then 2 min gradient to 4% B and finally an isocratic flow for 5 min at 4% B. After trapping, during the entire analytical run the solvent flow rate was set to 300 nL/min and the eluting peptides were separated on a downstream analytical column (Acclaim PepMap ® 100, 75 µm x 25 cm, particle size 3 µM, nanoViper, Thermo Fisher) kept at 35°C before being ionized by a Triversa Nanomate (Advion) ion source and entering the Orbitrap Q Exactive mass spectrometer: Q Exactive mass spectrometer was run in a TopN setting of 10 with data-dependant decision on precursor selection for fragmentation and MS2. Parameters set for MS1 were: Polarity =Positive, Default charge state =2, Microscan's =1, Resolution =120,000 Hz, AGC target =3,000,000 ions, Maximum injection time =100 ms, Scan range= 350 to 1600 m/z, Spectrum data type =Profile. Parameters for precursor selection and fragmentation were: Isolation window =1.4 m/z, normalized collision energy =28%, Underfill ratio =1.0%, Intensity threshold =20,000 ions, Charge exclusion =unassigned, 1, 7, 8 and >8, Peptide match =Preferred, Exclude isotopes =on, Dynamic exclusion =30.0 s. Parameters for MS2 were: Resolution =15,000 Hz, AGC target =200,000 ions, Maximum injection time =100 ms, TopN =10, Scan range =200 to 2000 m/z, Spectrum data type =centroid.

For mass spectrometers, the 445.12003 m/z internal lock mass was used for the measurements.

### Metaproteomics: bioinformatics, statistics and data analysis

Raw spectra data files were processed using Proteome Discoverer version 1.4 (Thermo Fisher Scientific, v1.4, San Jose, CA, USA). Spectra searches were performed by the tandem mass ion search algorithms Sequest HT. The genomic database for spectral searches was constructed by downloading and combining protein sequences from NCBI (National Center for Biotechnological Information, Rockville Pike, USA, August 2014) of all the bacteria genera identified in the 16S rRNA gene sequencing data with protein sequences from the taxa *Rattus norvegicus*, from two common archaea genera found in the gut and from plants commonly found in feed (Supplemental Table 1). Additional search parameters were tryptic cleavage, maximum of two missed cleavage sites, a precursor mass tolerance threshold of 10 ppm and a fragment mass tolerance threshold of 0.02 Da. Also, carbamidomethylation at cysteines was selected as a static and oxidation of methionine selected as a variable modification. Data were filtered with a peptide FDR set at 1% and only “rank one” peptides were considered for later analysis. Protein grouping was enabled. Label-free quantification of protein group intensities was calculated by averaging the peak areas of the top 3 peptides for protein group using the precursor ions area detector node of Proteome discoverer software.

The mass spectrometry proteomics data have been deposited to the ProteomeXchange Consortium via the PRIDE [[17](#_ENREF_17)] partner repository with the dataset identifier PXD013337.

PROteomics results Pruning & Homology group ANotation Engine” (PROPHANE) was used to assign proteins to their taxonomic and functional groups [[18](#_ENREF_18)]. For each protein group, the taxonomy annotation was based on the NCBInr protein database using BLASTP v2.2.28+ on all proteins binned to the protein group and only considering hits with an e-value ≤0.01. Functional prediction of protein groups was based on COG-database and KOG-database using RPSBLAST 2.2.28+ on all proteins from the protein group and only considering hits with an e-value ≤0.001.[[18](#_ENREF_18), [19](#_ENREF_19), [20](#_ENREF_20)]

Transforming, normalisation and statistical analysis of protein group intensity data were performed by an in-house written R script. The script first sums intensity values for each identified protein group from all fraction of the same sample. This was done by dividing the protein group intensities by the median of all the protein group intensities of the fraction and then multiplying by the median of these medians from all fractions of the same sample. Then these converted intensities were summed across all fractions for the sample if they came from the same protein group. These summed intensities were then log_10_ transformed and normalised by the transformed median intensities of the sample multiplied by the median of the log_10_ transformed medians of all samples from the same gut locality. Thus the different MS measurements were averaged. Only protein groups identified in at least three biological replicates from five in both conditions (RYGB and sham) were considered for relative quantification, replicates in which the protein group was not identified were considered as not detected. For log_10_ fold-change in protein group abundance, the mean normalised value from all biological replicates from the RYGB samples was subtracted from the mean normalised value from all biological replicates from the sham samples. These values were the label-free quantification (LFQ) values. For statistical analysis of fold changes, a two-sided independent student test was performed. For a protein group to be considered unique for one condition, it had to be identified in at least 3 replicates of that treatment and in none in the other treatment with *P* calculated using the Wilcoxon rank test. All *P* were corrected for multi-testing using the Benjamini-Hochberg method [[21](#_ENREF_21)].

Metaproteomic analysis results are based on either relative number of identified protein groups identified in the samples or on label-free quantification (LFQ) data (see above).

For taxonomic analysis by metaproteomics, only taxa which were identified in at least 3 replicates from 5 in one condition were further used for statistical analysis, as described above for single protein groups. For functional pathway analysis, KEGG [[22](#_ENREF_22)] as well as the Metacyc [[23](#_ENREF_23)] website applications were used. For calculation of significance of changes in metabolomics pathways adjusted *P* from the relative number of protein groups involved in the pathway, from the unique protein groups and from LFQ values of the relative quantifiable protein groups were combined and using the sum of *P* method (sump) [[24](#_ENREF_24)] implemented in the metap package from R.

### Metabolite extraction from samples

Metabolites were extracted from gut content samples by adding 5 µL H_2_O/Acetonitrile (1:1,v:v) per 1 mg of the sample then homogenising with a tissue slicer (10 min, 30 Hz, 4 steel balls). This was followed by sonication (5 min) Samples were centrifuged (14,000 g, 2 min) and the supernatant was aliquoted for targeted and untargeted metabolomics and kept at -80°C. Serum samples were frozen directly stored without preparation at -80°C.

### Targeted metabolomics

The targeted analysis was performed by adding 10 μL of the extracted metabolome (cecum: RYGB n=9 and Sham-BWM n=4; colon: RYGB n=7 and Sham-BWM n=7) or serum samples (RYGB n=6; Sham-BWM n=5) to the Absolute*IDQ*® p180 Kit (Biocrates Life Science AG, Innsbruck, Austria), following the vendor's instructions. The Kit identifies and quantifies 186 metabolites from 5 compound classes, i.e., acylcarnitines (40), amino acids (19 proteinogenic, citrulline and ornithine), glycerophospho- and sphingolipids (76 phosphatidylcholines, 14 lysophosphatidylcholines, 15 sphingomyelins), biogenic amines (19) and hexoses [[25](#_ENREF_25)].

For LC-MS analysis of biogenic amines and amino acids and flow injection analysis-MS/MS measurements (FIA-MS/MS) were used for two different dilutions. Both types of measurements were performed on a QTRAP mass spectrometer applying electrospray ionisation (ESI) (ABI Sciex API5500Q-TRAP). The MS was coupled to a UPLC (Waters Acquity, Waters Corporation, Milford, USA). In case of LC-MS the metabolites were separated by a hyphenated reverse phase column (Agilent, Zorbax Eclipse XDB C18, 3.0 x 100 mm, 3.5 µm , Agilent Waldbronn, Germany) preceded with a precolumn (Security Guard, Phenomenex, C18, 4 x 3 mm; Phenomenex, Aschaffenburg, Germany) applying a gradient Identification and quantification were achieved by multi-reaction monitoring (MRM) standardized by applying spiked-in isotopically labelled standards in positive and negative mode, respectively. For calibration, a calibrator mix consisting of 7 different concentrations was used. Quality controls were included for 3 different concentration levels. For FIA an isocratic method was used. The integrated MetIDQ software (Biocrates, Innsbruck, Austria) streamlines data analysis by automated calculation of metabolite concentrations providing quality measures and quantification [[26](#_ENREF_26)].

For bile acid measurements the Bile Acids Kit (Biocrates Life Sciences AG, Innsbruck, Austria), a 96-well plate format assay, was used as described in the manufacturer´s instructions. In short, 10 µL of internal standards mixture was pipetted onto the filter spots suspended in the wells of the 96-well filter plate. This filter plate was then fixed on top of a deep-well plate serving as a receiving plate for the extract later on, i.e. a combi-plate structure. After a short drying under a nitrogen stream, 10 µL samples were pipetted, followed again by the nitrogen drying. 100 µL of methanol was then added to the wells, and the combi-plate was shaken for 20 min on a plate shaker. The combi-plate was then centrifuged to gain the methanol extract into the lower receiving deep-well plate, which then was detached from the upper filter plate. 60 µL Milli-Q® water was added to the extract. The plate was placed in the autosampler of the LC-MS/MS system for analysis after a short shaking. Target bile acids are chromatographically separated on a reversed phase column. All isobaric bile acids can be baseline separated under both HPLC and UHPLC condition using the same column. Under UHPLC conditions a higher flow rate of 0.5 min/min was employed with a runtime of 5 min. Under the HPLC conditions, a lower flow rate of 0.4 mL/min has to be used to keep the column pressure under the pressure limit, which is 400 bar on most of the HPLC systems. The HPLC runtime is, therefore, prolonged to 11 min. The triple quadrupole mass spectrometer (MS/MS) was operated with an electrospray source in negative mode. Due to the facts that most of the bile acids, especially the unconjugated ones, do not fragment well, the most intensive signals were obtained with daughter ions having the same mass as the parent ions. This signal was used as a quantifier. It differs from the purely selected ion monitoring (SIM) mode in the fact that certain collision energy still applies in the collision cells to detect the signals. A weaker signal, for example, a transition from the parent ion to the glycine or taurine moiety in case of glycine or taurine conjugates, respectively, was used as the qualifier. For the quantitation, a calibration set with seven concentrations levels and a mixture of 10 internal standards were used [[26](#_ENREF_26)]

### Untargeted metabolomics

For each sample, 100 µL metabolite containing metabolome extract (cecum: RYGB n=8 and Sham-BWM n=4; colon: RYGB n=8 and Sham-BWM n=7) or 100 µL serum, was mixed with 500 µL MeOH/ Acetonitrile /H_2_O (3:2:1; v:v:v). Mixtures were vortexed for 5 min, sonicated for 5 min and spun at 14,000 rcf for 5 min. 550 µL of supernatant for each sample was transferred into a glass vial and liquid evaporated in a speed vac at 45°C. Samples were resuspended in 100 µL H_2_O/Acetonitrile (1:1; v:v) solvent. 20 µL of each sample was separated on a LC-QTOF-MS 6540 from Agilent Technologies (Waldbronn, Germany) by reverse phase-LC (RP-LC) on a C18 column (Acquity UPLC HSS T3, 2.1x100 mm from Waters GmbH, Eschborn, Germany) with a gradient of water and acetonitrile (both containing 0.1% of formic acid) [[27](#_ENREF_27)]. The gradient started with 1% of acetonitrile for 5.5 min and increased up to 100% acetonitrile in 50 min. After 5 min the composition of acetonitrile decreased back to the original conditions in 5 min and stayed constant for 5 min. Eluting analytes were measured on the QTOF-MS in full scan mode (detecting masses from 60 to 1000 m/z). For each sample two MS measurements were performed, one in positive ion mode and one in negative ion mode [[28](#_ENREF_28)]. For the data treatment, the same protocol as used by Samino *et al.* [[29](#_ENREF_29)] with minor modifications was followed: raw data files (.mzdata format) were loaded to XCMS online [[30](#_ENREF_30)] to perform the peak picking, grouping of similar peaks, and retention time alignment. Then, only the peaks appearing in at least 80% of the replicates of one condition and above the intensity threshold of 2000 counts (which is the intensity above which the ions are sent to the second MS for fragmentation) were selected for the statistical analysis. Nonparametric Multi Dimension Scaling (NMDS) of the selected peaks was carried out by using an R script developed in-house.

### Network analysis

Co-occurrence and co-excluding networks among taxa, metaproteomic pathways and metabolite classes were identified by SparCC algorithm [[31](#_ENREF_31)]. Firstly, sub-networks were calculated for separated datasets respectively (different intestinal segments, RYGB or Sham). Different cut-offs of significance level were applied: for the links between two taxa: SparCC coefficient > 0.9 or < -0.9, *P* < .01; for links between metaproteomic pathways, metabolite classes and taxa: coefficient > 0.8 or < -0.8, *P* < .05. Thus, the intra-segment networks were constructed. Secondly, to associate different items among different intestinal segments, supplemental networks were built by combining all data from all intestinal segments for each surgery type respectively (RYGB or Sham). The inter-segment cut-offs of significance level were set as: between two taxa from different intestinal segment: coefficient > 0.9 or < -0.9, *P* < .001; for links between metaproteomic pathways, metabolite classes and taxa: coefficient > 0.8 or < -0.8, *P* < .05. By combing the intra-segment edges from the first step and the inter-segment edges of the second step, co-occurrence and co-excluding networks were built by RYGB and Sham samples respectively.

The results were illustrated by CytoScape 3.5 [[32](#_ENREF_32)]. The nodes of the items with significantly different abundances were filled with different colours (orange for RYGB and blue for Sham). Different border colours represented the items with a significantly higher abundance in one of the three intestinal segments (blue for Cecum, purple for colon content and orange for ileum content). Node size represented the z-score of an item’s relative abundance. Edge line types illustrated different association items (intra- or inter-segment, between taxa or between taxa and functions). Edge width and colour represented the correlation co-efficient and direction

Bile acid and arginine metabolism specific network analyses were carried out separately for cecum and colon content with Cytoscape plugin CoNet [[33](#_ENREF_33)]. Only proteins with COG clusters associated with arginine pathway were used as an input for calculation of arginine networks. For calculation of bile acid networks, only proteins from taxa, which are known to be associated with bile acids pathways were used as an input matrix. Additionally, metabolites of interest were used as an second input matrix for all networks (arginine pathway metabolites: Arg, Asp, Cit, Orn, Ac-Orn; bile acids: CA, CDCA, DCA, GCA, GCDCA, GDCA, GLCA, GUDCA, HDCA, LCA, MCA(a), MCA(b), MCA(o), TCA, TCDCA, TDCA, TLCA, TMCA(a+b), TUDCA). Network calculation was carried out with the following parameters: Pearson and Spearman correlation, as well as Bray-Curtis and Kullback-Leibler dissimilarity as methods. Proteins which were not present in at least 60 % of the samples were discarded for the arginine pathway (bile acids: 80 %). The threshold was set to automatic and each method contributed 1,500 positive and 1,500 negative edges for the initial arginine network (3,000 positive and 3,000 negative edges for bile acids, respectively). Only edges supported by at least two of the four methods were kept. The measure-specific p-values were then computed by using the mean and standard deviation of the bootstrap distribution (100 iterations) as a parameter of the normal distribution. Measure-specific *P* were then merged using the method of Brown [[34](#_ENREF_34)] taking correlation among measures into account. Only edges with *P*< .05 were kept after multiple-testing correction of Benjamini and Hochberg [[21](#_ENREF_21)]. Nodes were assigned to modules by using GLay community algorithm [[35](#_ENREF_35)]. Finally, within-module connectivity (z) and among-module connectivity (P_i_) were calculated as described by Guimera and Amaral [[36](#_ENREF_36)] with automated in-house excel sheets. Peripheral nodes (specialists) were defined by z ≤ 2.5 and P_i_ ≤ 0.62, connectors by z ≤ 2.5 and P_i_ > 0.62, module hubs by z > 2.5 and P_i_ ≤ 0.62 and network hubs by z > 2.5 and P_i_ > 0.62.

## Supplemental Tables and Figures

Supplemental Table 1: Taxa used for metaproteome data base


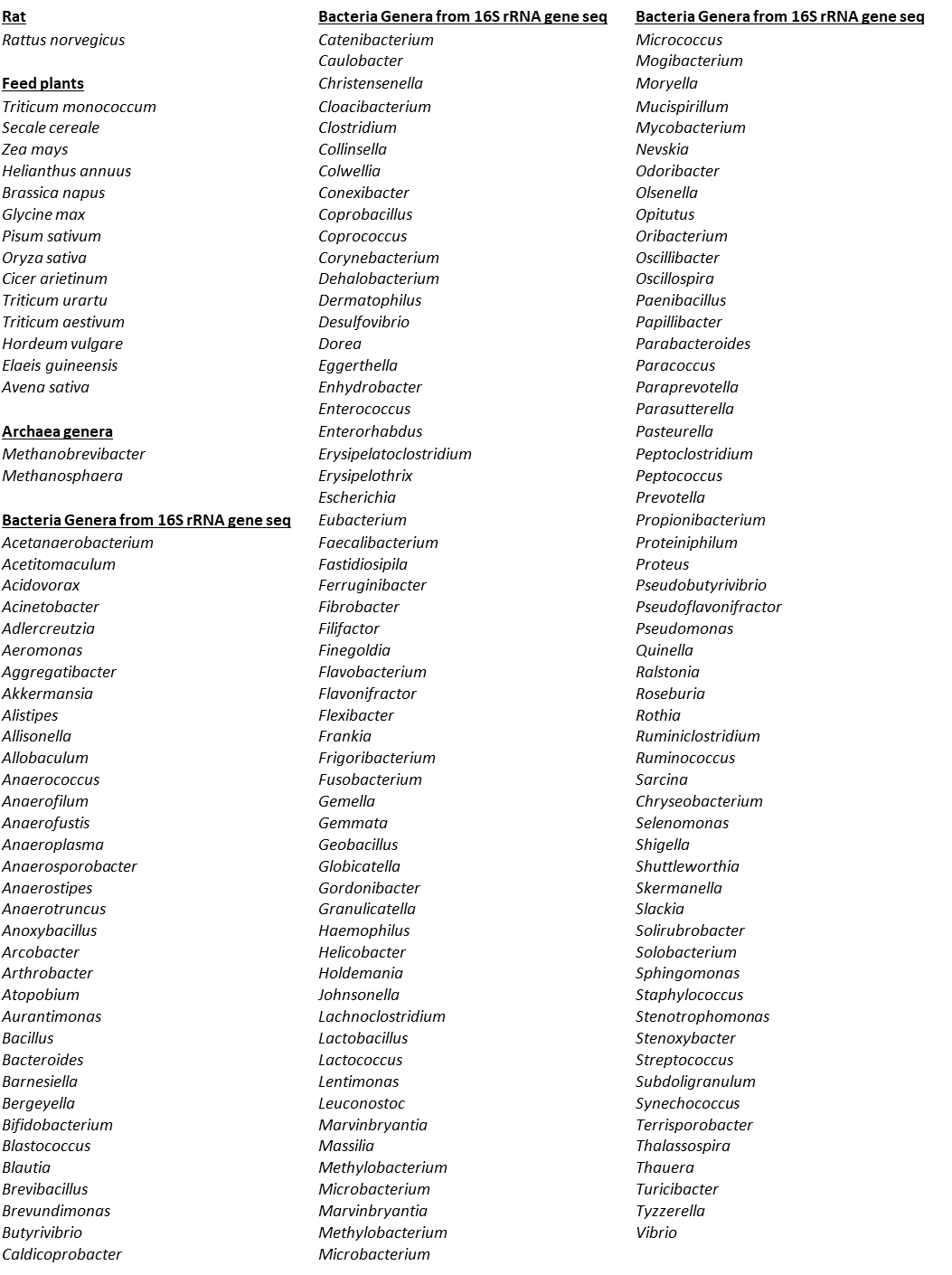


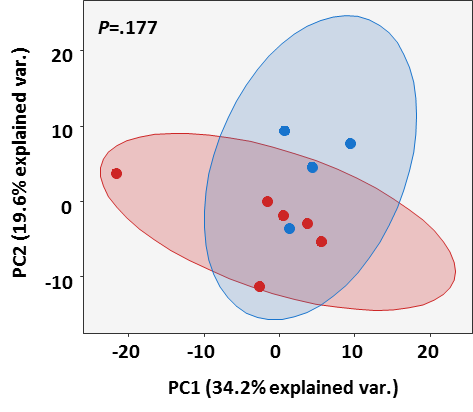


Supplemental Figure 1: Global analysis of targeted blood serum metabolomics by Principal Component Analysis (PCA). Significance calculated by PERMANOVA using the Adonis function in vegan package for R. (RYGB samples = red dots, Sham-BWM samples = blue dots).


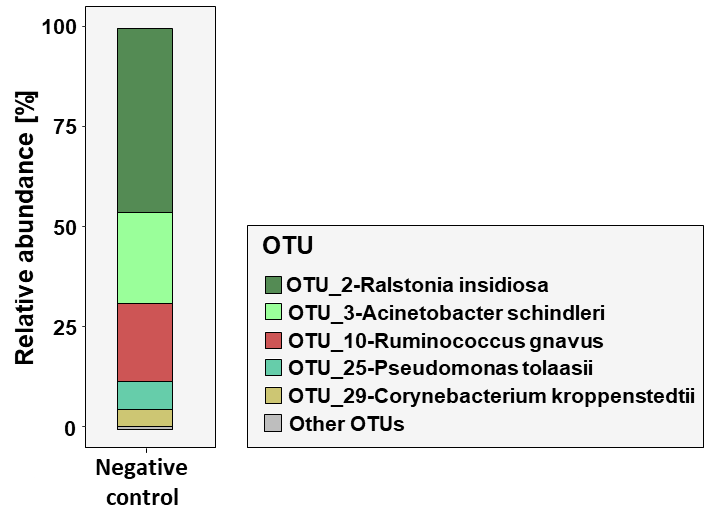


Supplemental Figure 2: Relative distribution of taxa based on reads from 16S rRNA sequencing data from the negative control.


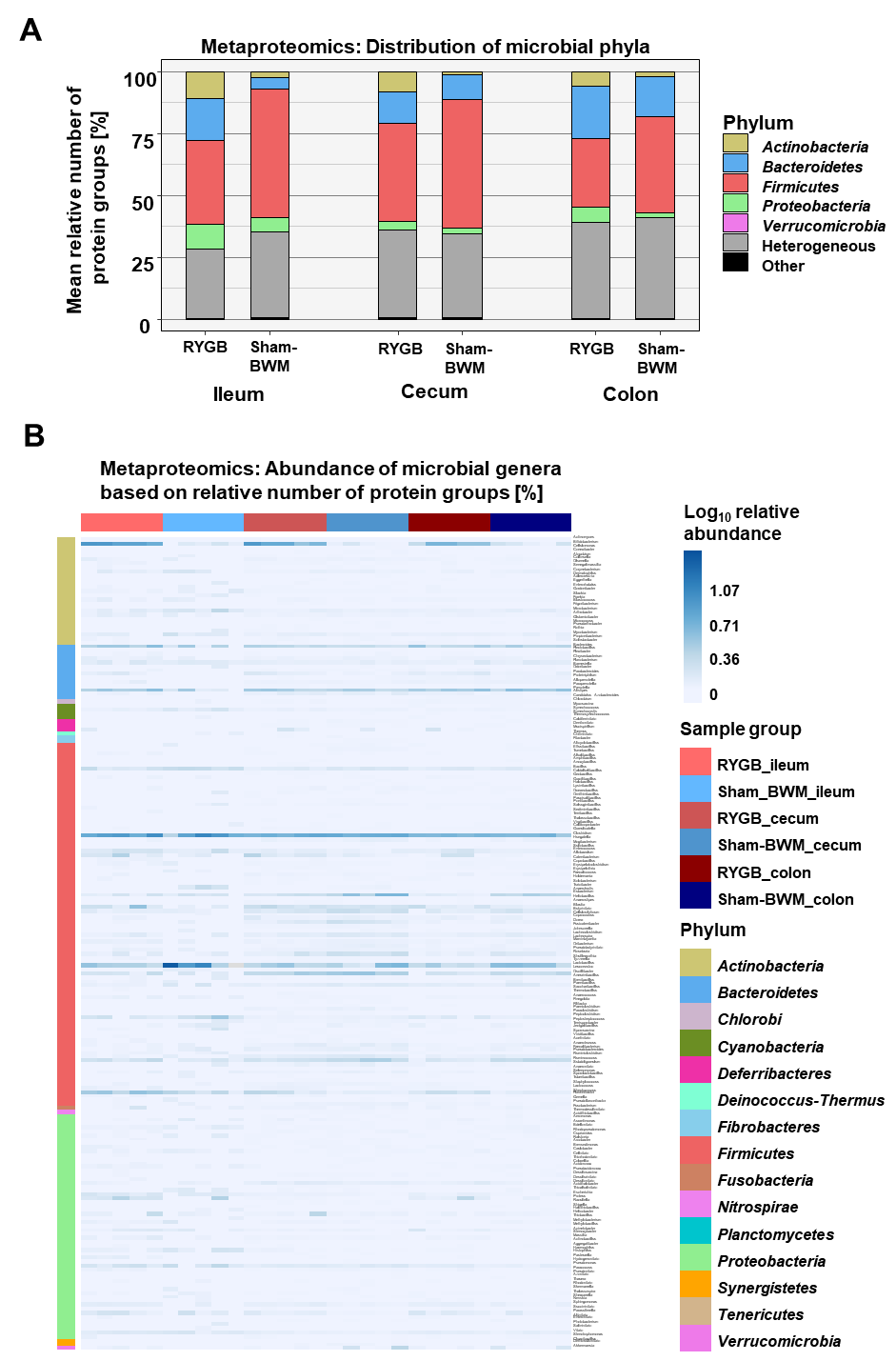


Supplemetal Figure3: Relative abundance of microbial phyla (A) and genera (B) based on metaproteomic data


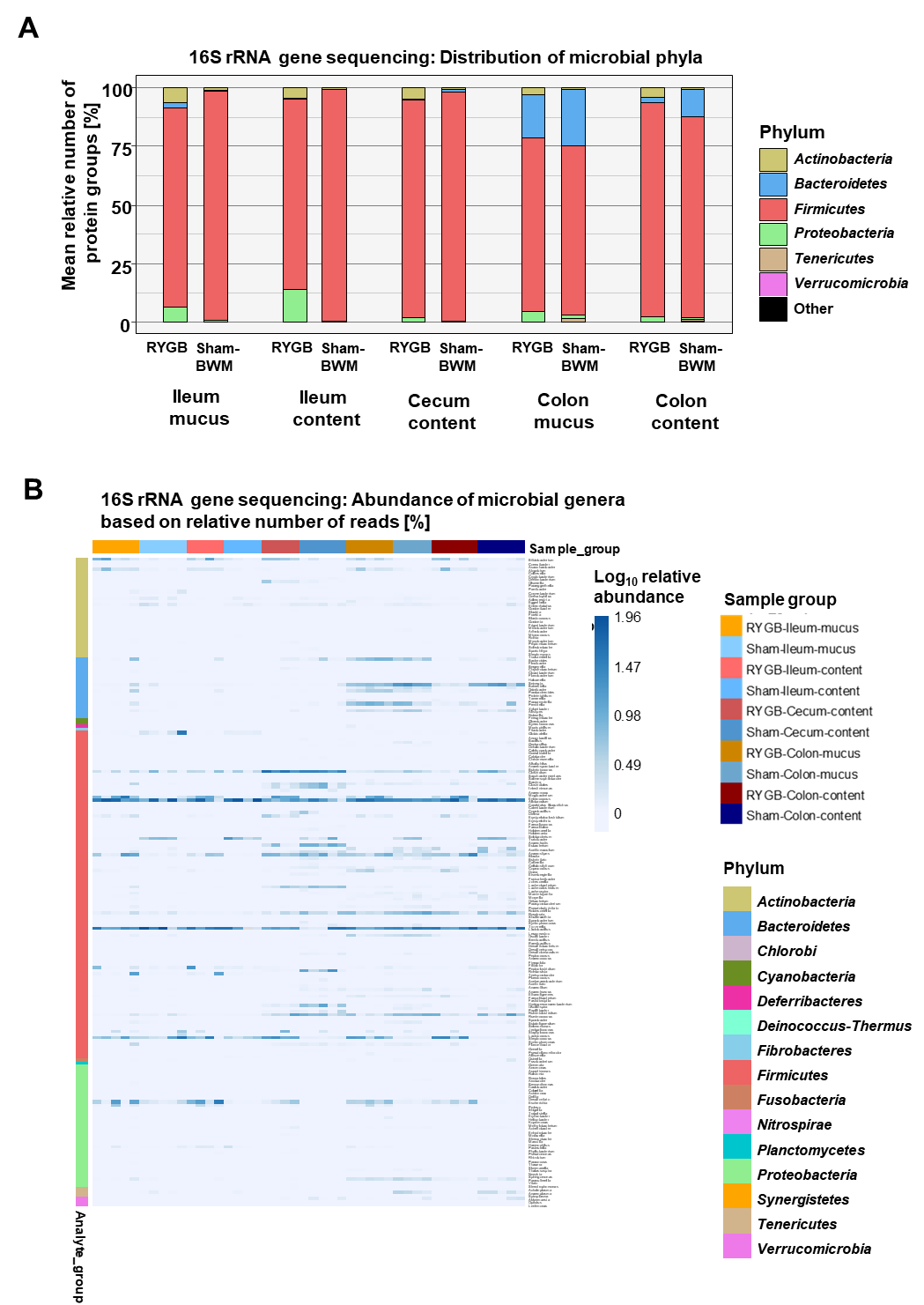


Supplement Figure 4: Relative abundance of microbial phyla (A) and genera (B) based on 16S rRNA gene sequencing data


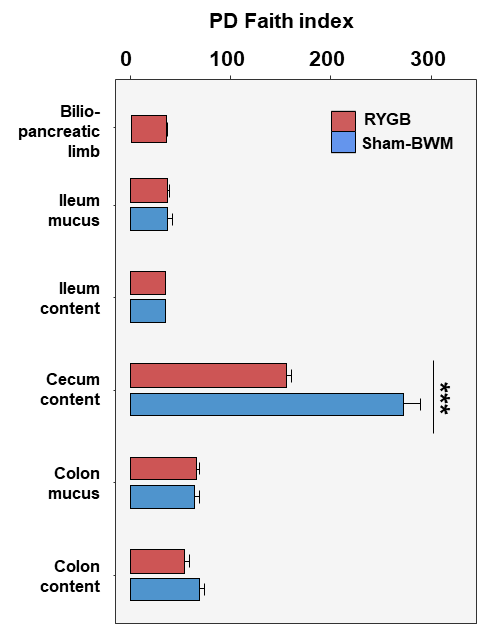


Supplement Figure 5: Alpha diversity, mean PD Faith indices of 16S rRNA gene sequencing data. (*** = *P*<.0001; error is SEM)


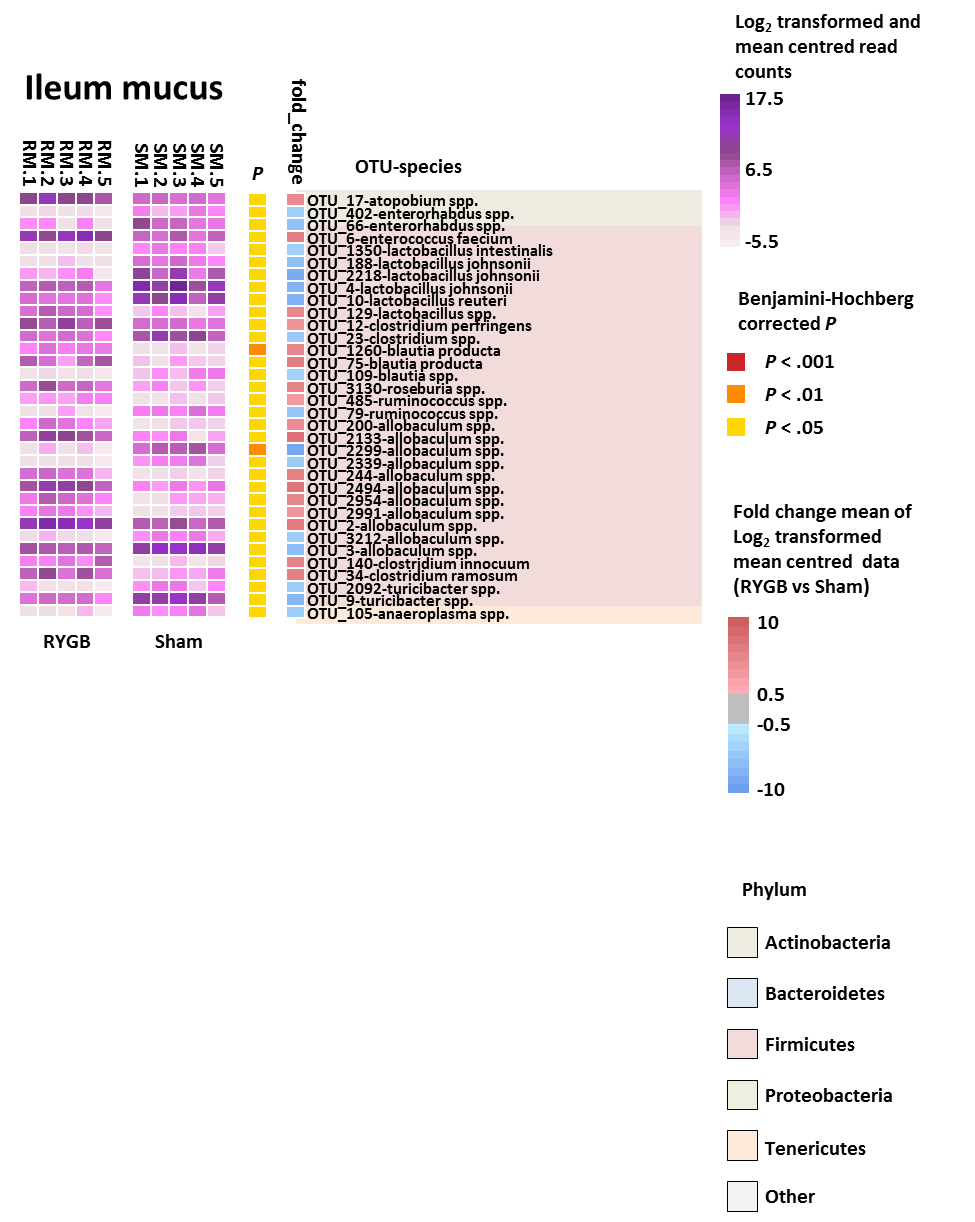


Supplemental Figure 6: Significant changes in OTUs abundances based on 16S rRNA gene sequencing data observed in the ileum mucus.


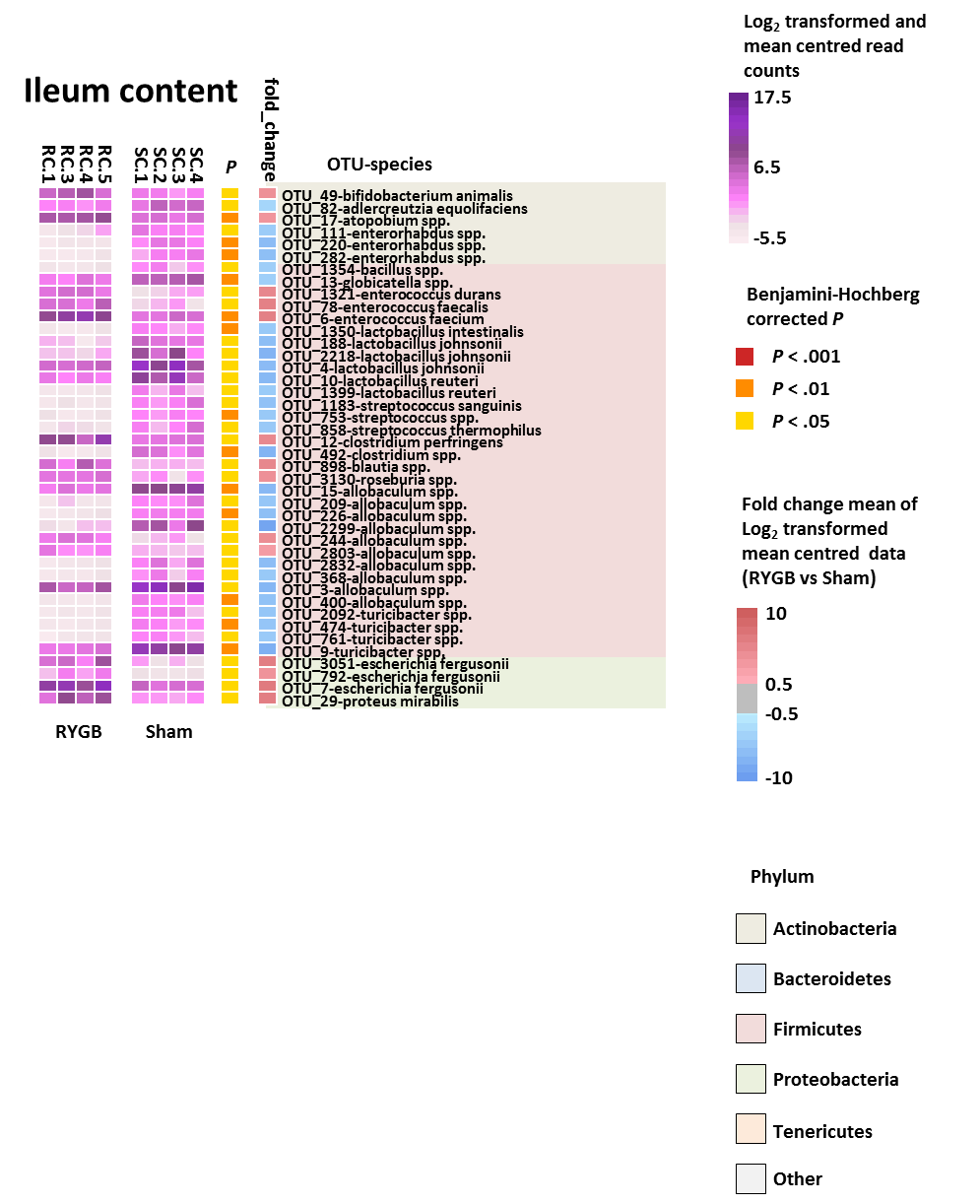


Supplemental Figure 7: Significant changes in OTUs abundances based on 16S rRNA gene sequencing data observed in the ileum content.


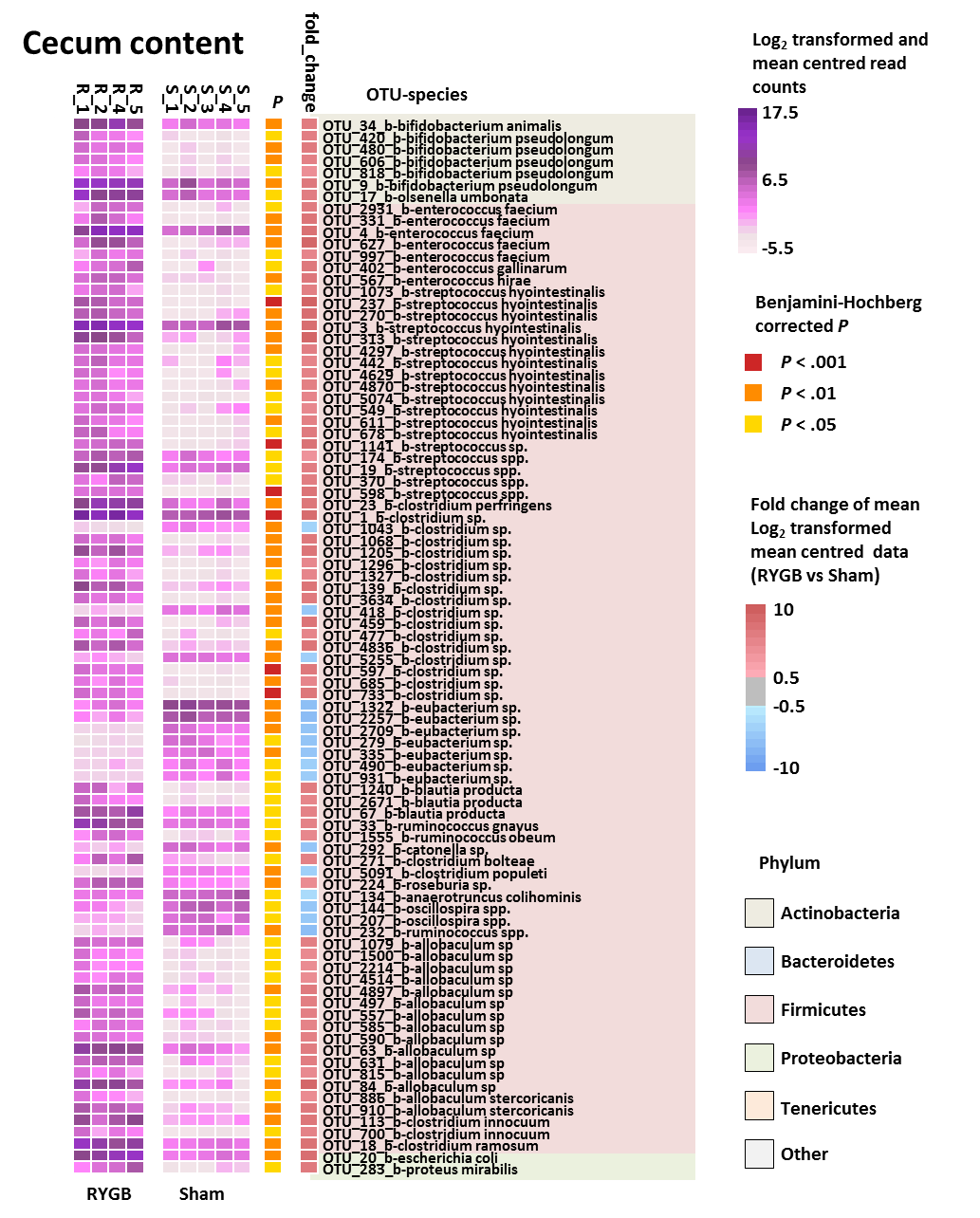


Supplemental Figure 8: Significant changes in OTUs abundances based on 16S rRNA gene sequencing data observed in the cecum content.


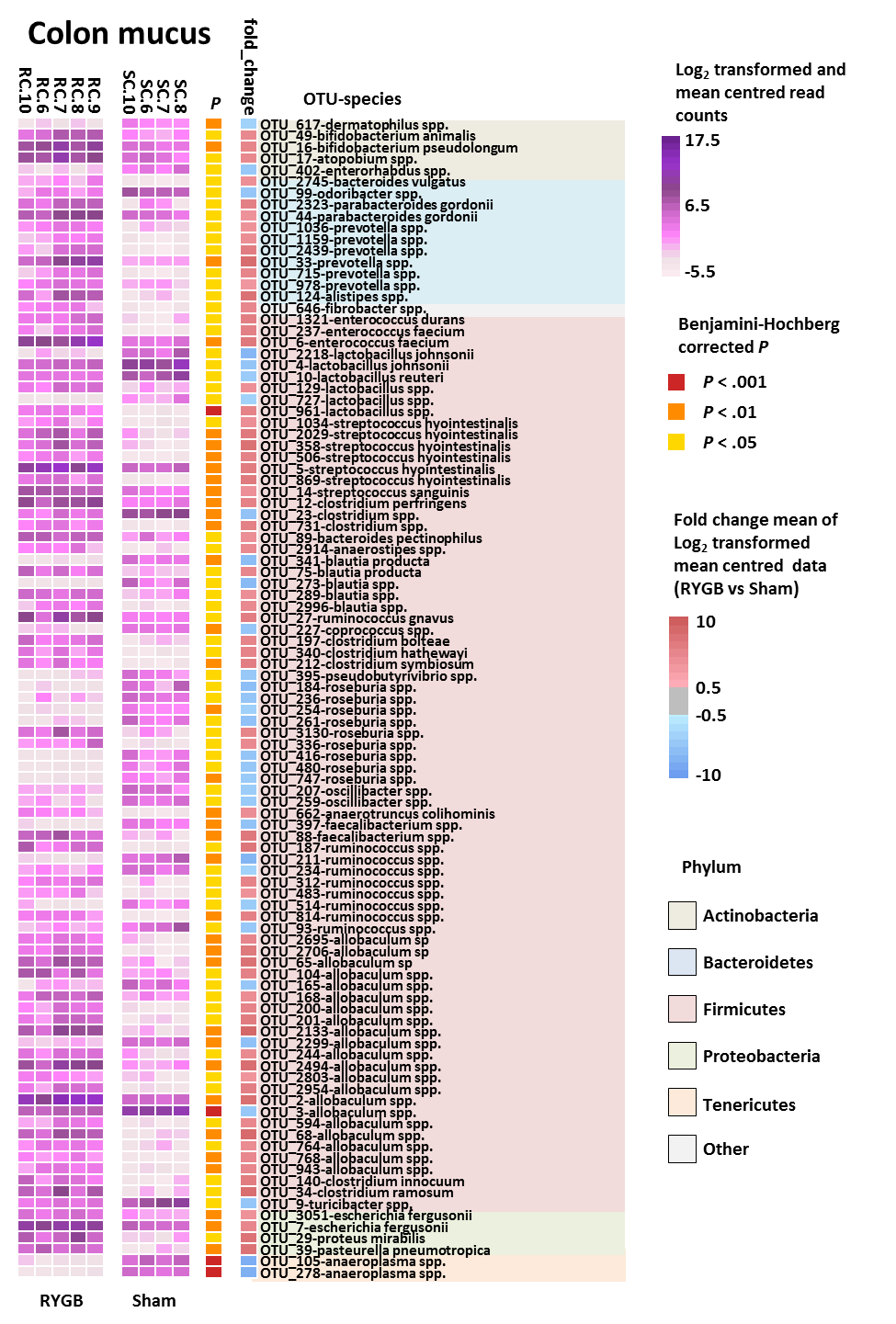


Supplemental Figure 9: Significant changes in OTUs abundances based on 16S rRNA gene sequencing data observed in the colon mucus.


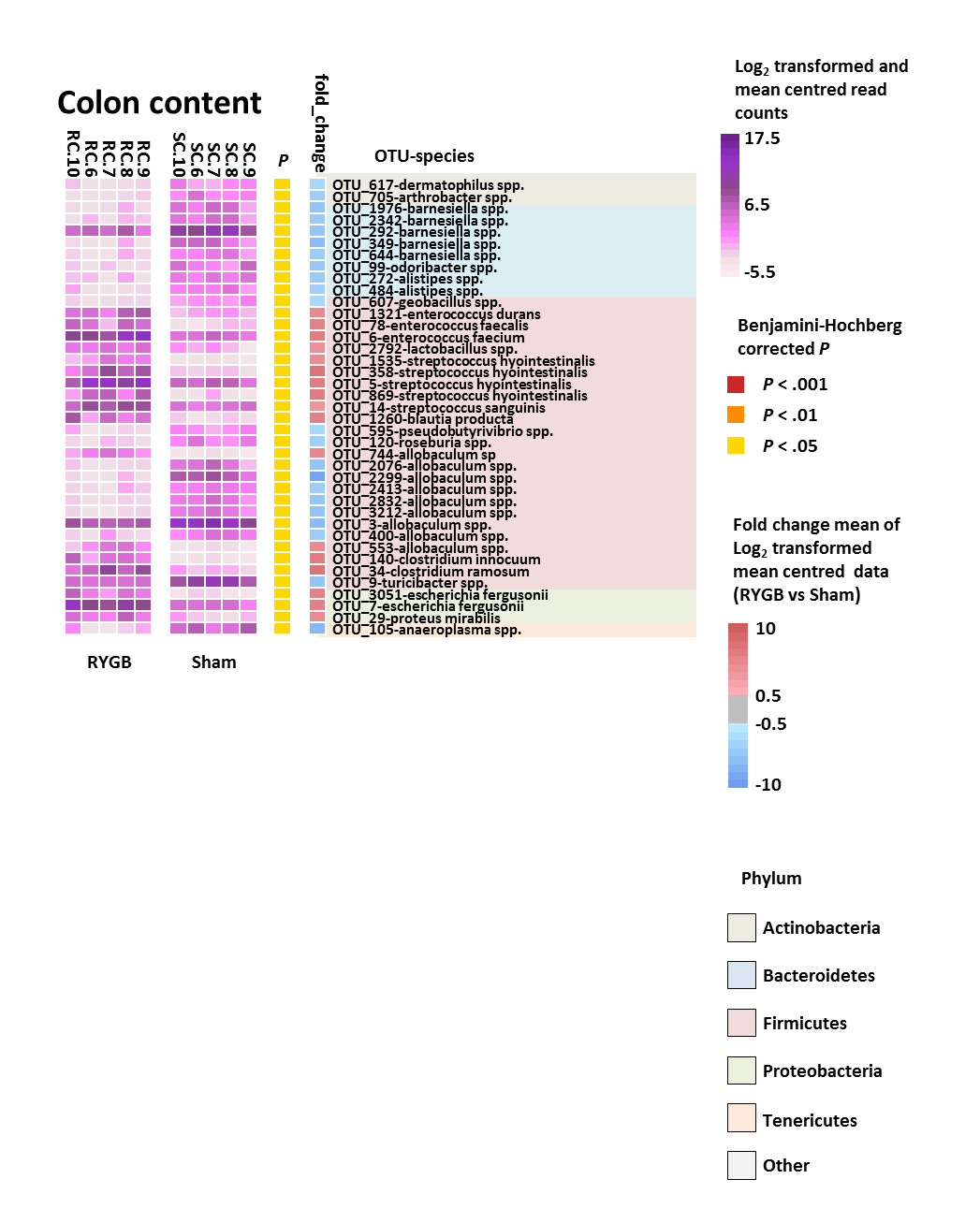


Supplemental Figure 10. Significant changes in OTUs abundances based on 16S rRNA gene sequencing data observed in the colon content.


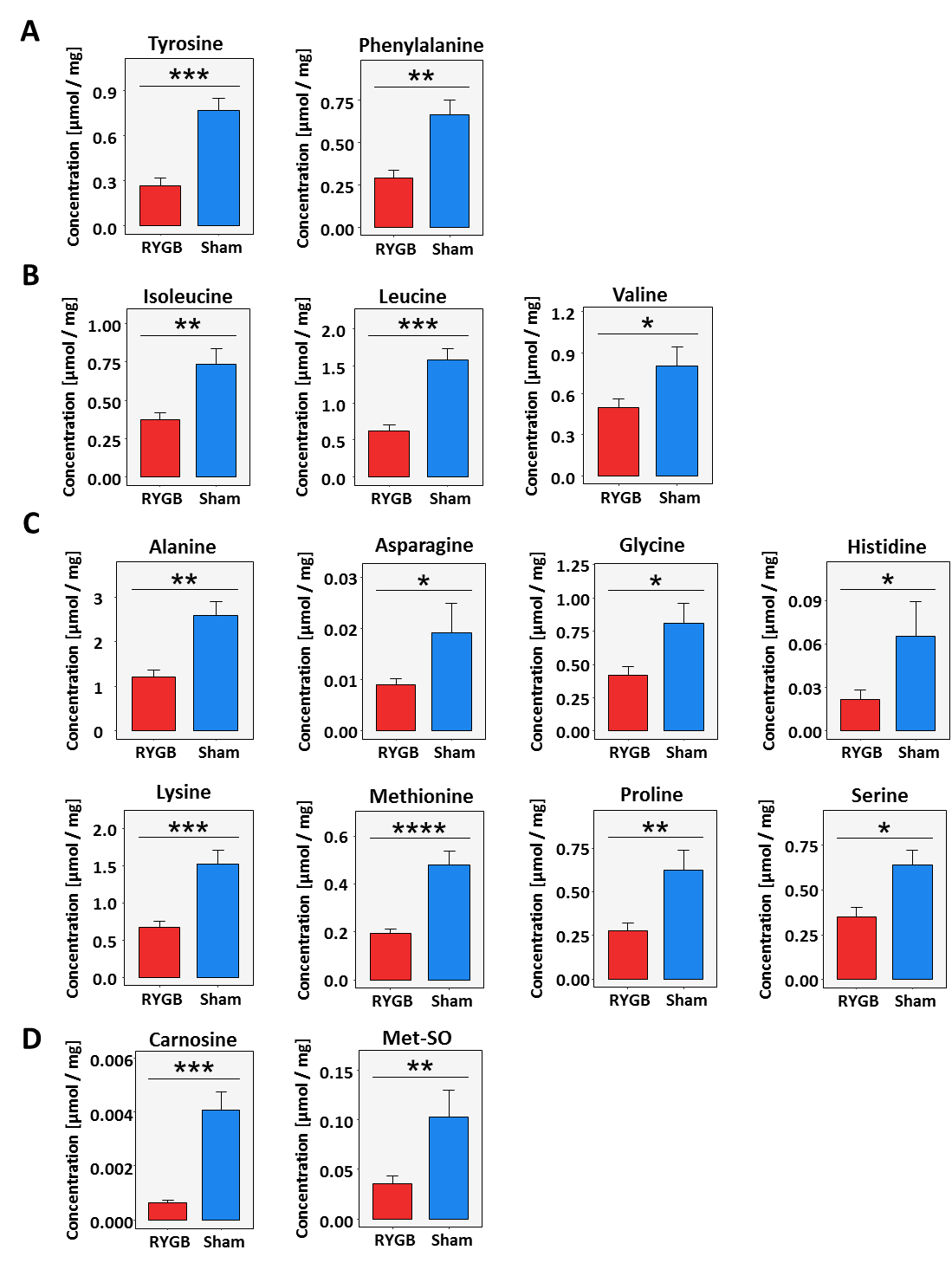


Supplemental Figure 11: Metabolomic data from the cecum content (RYGB n=9, Sham-BWM n=4). A: Aromatic amino acids B: Branched chain amino acids C: Other amino acids D: Amines. Error bars depict standard error of mean (**** = *P* <.0001, *** = *P* <.001, ** = *P* <.01, * = *P* <.05).


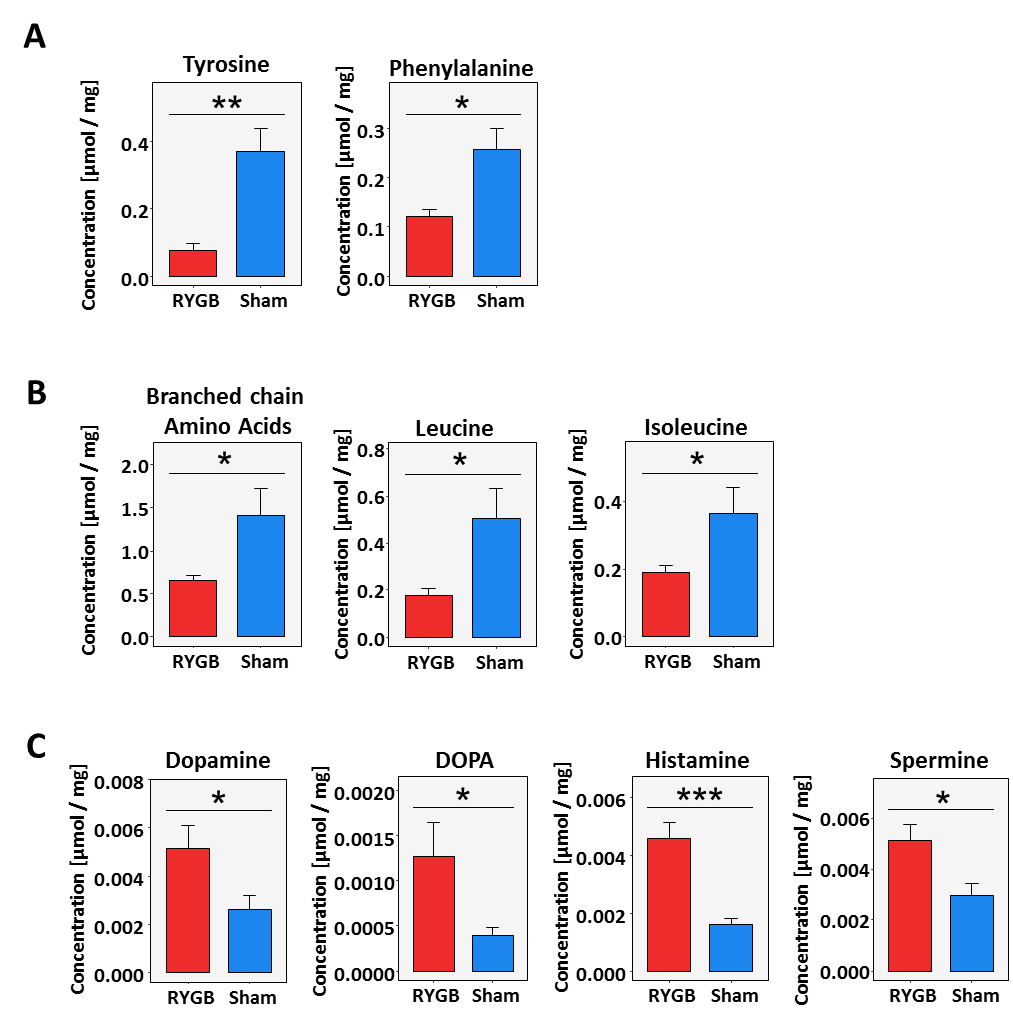


Supplemental Figure 12: Metabolomic data from the colon content (RYGB n=7; Sham-BWM n=7). A: Aromatic amino acids B: Branched chain amino acids C: Amines. Error bars depict standard error of mean (**** = *P* <.0001, *** = *P* <.001, ** = *P* <.01, * = *P* <.05).


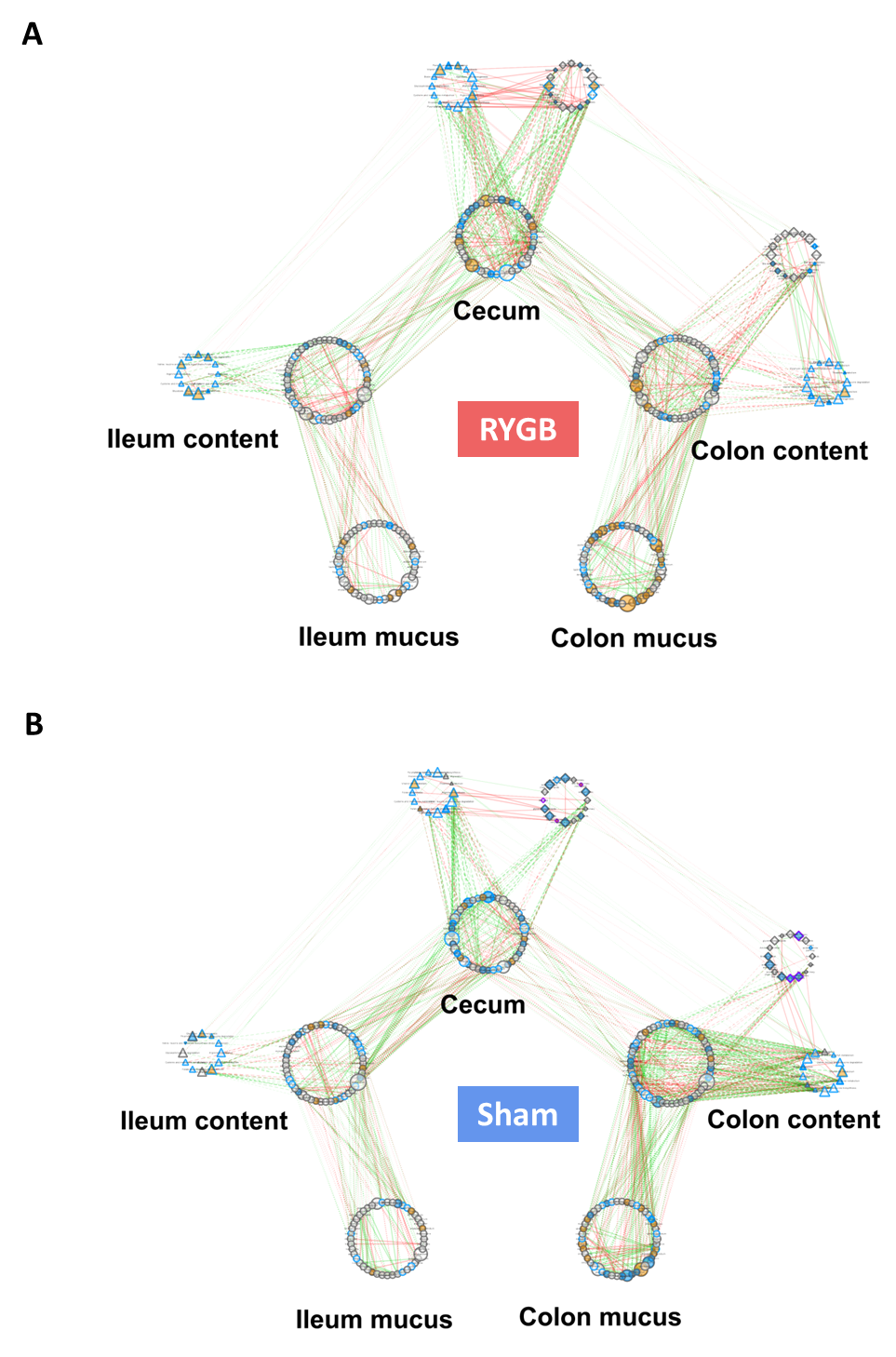


Supplemental Figure 13. Correlation network analysis of Taxa from 16S rRNA sequencing data (circles), functional pathways from metaproteomic data (triangles) and metabolites (diamonds). Red edges are positive and green edges are negative correlation.


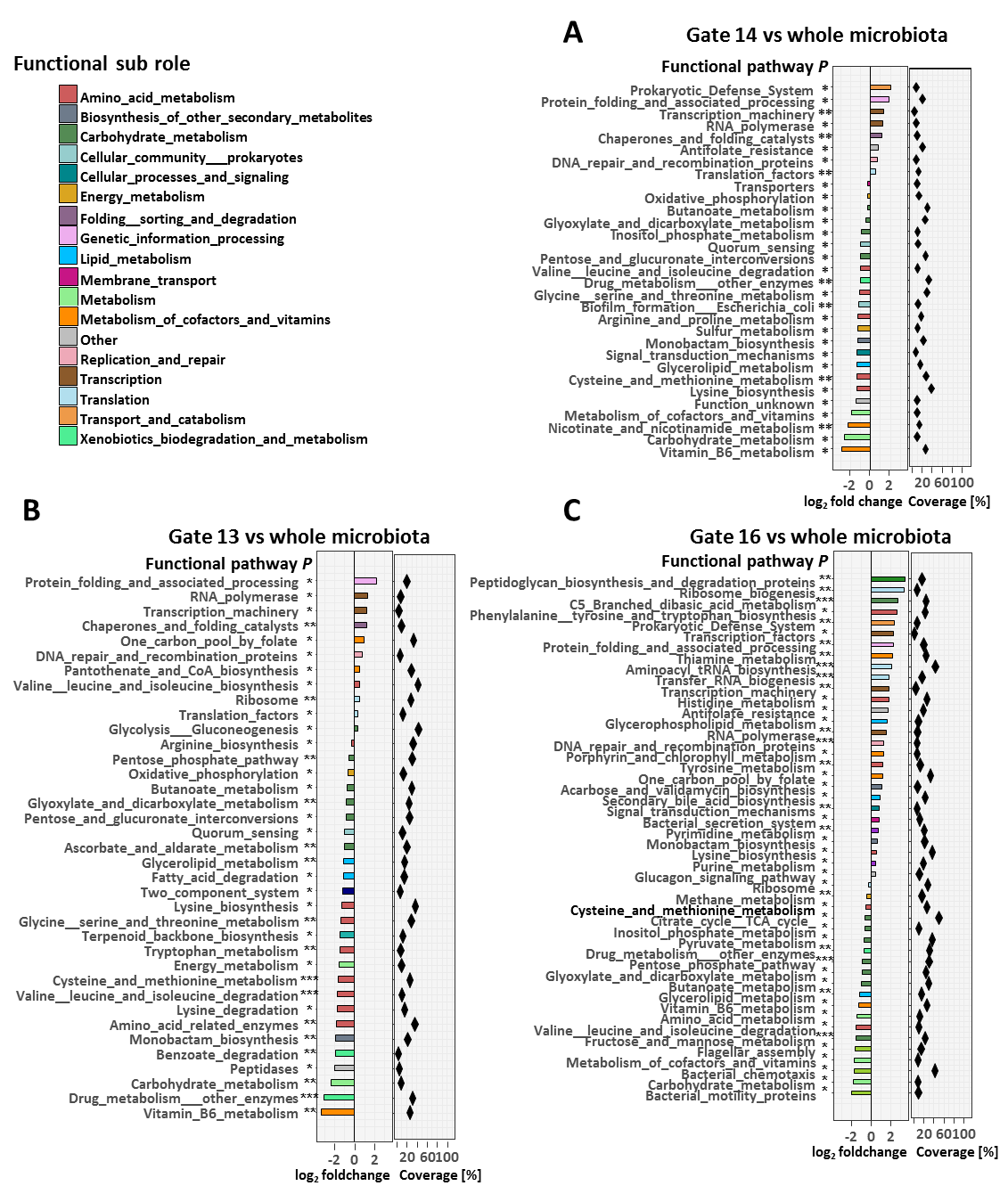
 **Supplemental Figure 14: Comparison of sorted bacterial cells from RYGB cecum content (n=4) with the entire microbiota from RYGB cecum content (n=5). A: significant changes in bacterial families based on relative number of protein groups. Significant altered enrichment of functional pathways for Gate 14 (B), Gate 13 (C) and Gate 16 (D) (**** = P <.0001, *** = P <.001, ** = P <.01, * = P <.05)**

t**
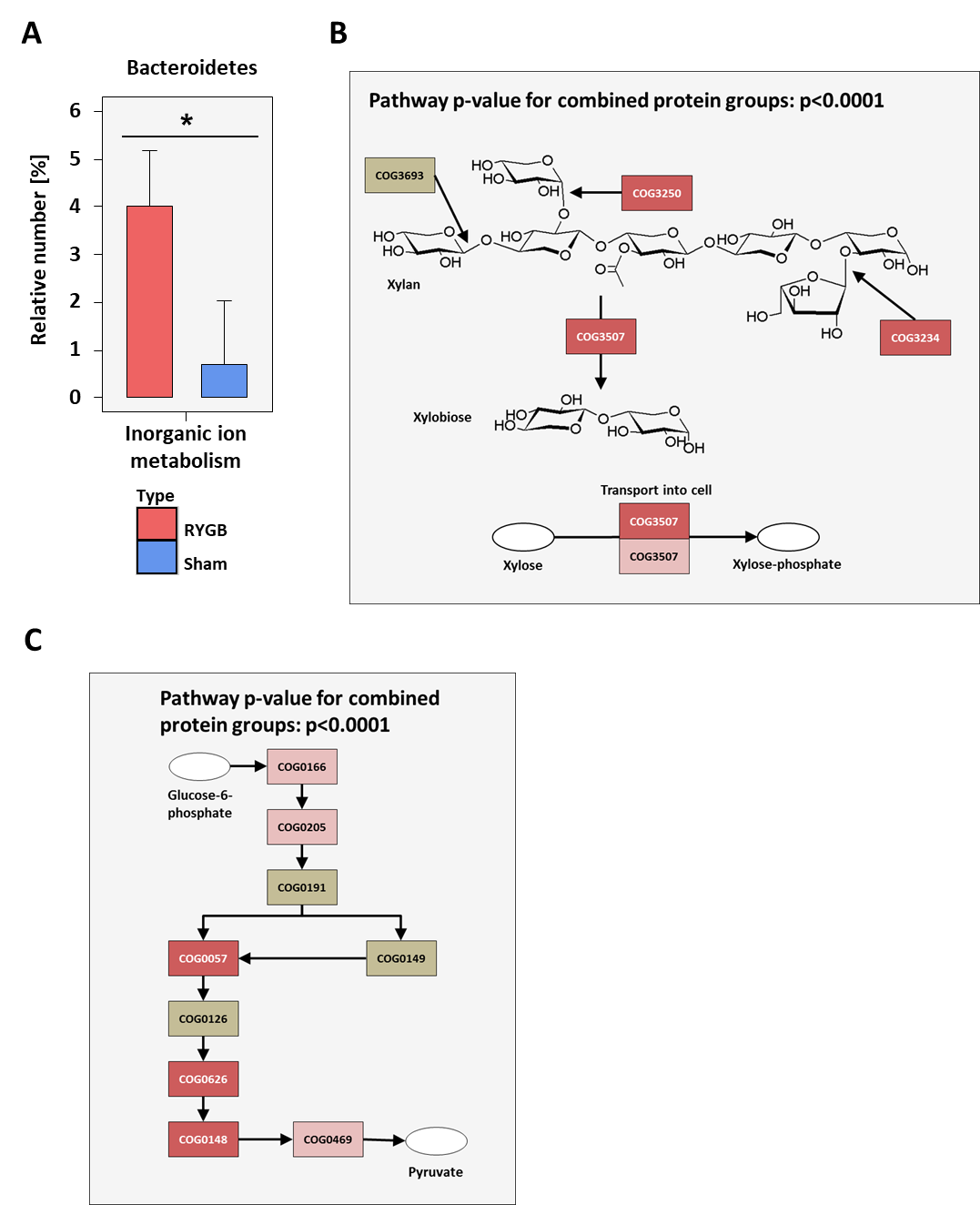
**

Supplemental Figure15: Metaproteomic functional data (**** = *P* <.0001, *** = *P* <.001, ** = *P* <.01, * = *P* <.05). A: relative number of protein groups from Bacteroidetes assigned to inorganic ion metabolism from the ileum. B: Protein functions involved in xylan utilization in the cecum content. C: Protein functions of the glycolysis. For pathway maps: Filled rectangles with rounded edges protein functions with Cluster of Orthologous Groups code with red greater presence in RYGB, pink some evidence of greater presence in RYGB, dark blue greater presence in sham, light blue some evidence of greater presence in sham, khaki protein function detected. Selected metabolites white ellipses. Red ellipsis at significantly higher concentrations in RYGB, blue ellipsis at significantly higher concentrations in sham, khaki ellipsis non-significant.


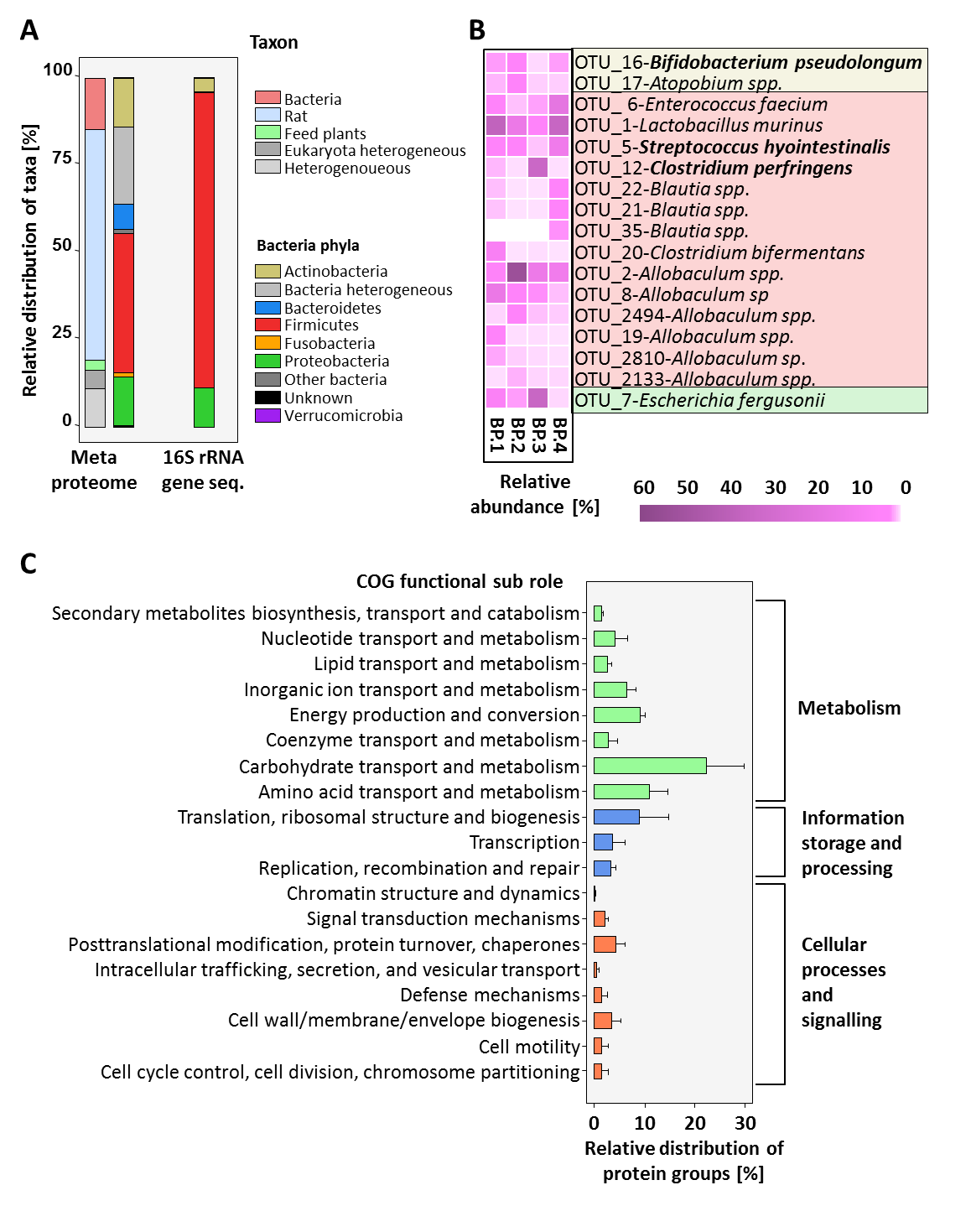


Supplemental Figure16: Description of the Biliopancreatic limb contents. A: Taxa distribution in biliopancreatic limb based on mean relative number of protein groups from metaproteomics (left all protein groups and right bacterial protein groups) and mean relative number of reads from 16S rRNA gene seq. B: Most abundant OTU’s identified in the biliopancreatic limb (mean relative abundance > 0.5%). C: Distribution of bacterial protein groups based on Cluster of Orthologous Groups (COGs) functional subroles. Error bars depict standard deviation.

## Supplemental Results

### Changes in animal body weight and host phenotypical parameters

Body weight of sham body weight-matched animals (sham) was kept at a similar level of RYGB-animals post-surgery (Figure 1). There were no significant differences in body weight between the RYGB and body weight matched sham (Sham) animal’s post-operative after 5 weeks. For confirming the stability on the host side we performed a targeted analysis of serum metabolites and found only very few changes among the 180 measured metabolites (Supplemental Figure 1). None of the amino acids or biogenic amines were at significantly higher or lower abundances in RYGB. There were only very few significant changes in the different lipid groups, but those were all metabolites that occur at very low concentrations.

### Sequencing data

After rare fraction curve analysis of the 55 samples sequenced, 5 samples were discarded from the analysis because of a lack of reads. These were one biliopancreatic sample, one RYGB as well as one sham ileum content sample, one RYGB cecum content sample and one sham colon mucus sample. In the small intestine, the Miseq 16S rRNA gene sequencing resulted for the biliopancreatic limb samples in 75,249 +-5,306 reads, for the ileum mucus in 78,921 +-7843 reads for RYGB and 65,950 +-12,243 reads for sham as well as for the ileum lumen content 77,747 +-7,130 reads in RYGB and 77,493 +-2716 reads for sham. In in the cecum, sequencing resulted in 140,779 +-12,822 reads for RYGB and 89,567 +-13,794 reads for sham. In the colon, in the mucus samples for RYGB, 65,213 +-8,564 reads and for sham 45,900 +-5,142 reads were detected whereas in the lumen content 53,248 +-3889 reads in RYGB and 50,199 +-6,541 reads for sham were observed. The negative control yielded 25,095 reads which were clustered into 35 OTU’s of which most were classified as Proteobacteria (Supplemental Figure 2). Most of the distribution in the negative control was attributed to only three OTU’s, and these were classified as *Ralstonia insidiosa* (relative abundance 45.7%)*, Acinetobacter schindleri* (22.7%) and *Ruminococcus gnavus* (19.6%).

### Metaproteomics

In total, for the biliopancreatic limb samples (n=4) 3,199 non-redundant protein groups were identified (mean 1,795 +-163). The ileum content samples (n=5) yielded a total of 6,496 non-redundant protein groups identified (RYGB: mean 2,498 +-596; Sham mean 1,468 +-582) of these 673 were relative quantifiable, 239 were unique for RYGB, and 726 were unique for sham. The cecum content (n=5) yielded in total 12,570 non-redundant protein groups (RYGB: mean 4,560 +-283; Sham: mean 4,258 +-395), of which 2,052 were relative quantifiable and 492 were unique for RYGB whereas 938 were unique for sham. The colon content (n=5) yielded 8,985 (RYGB: mean 3,693 +-440; sham: mean 3,401 +-157) protein groups with 2,200 relative quantifiable as well as 430 unique for RYGB and 737 unique for sham. For the cecum sorted samples (each gate n=4) the number of identified protein groups were for gate 13: 934 +-176, for gate 14: 687 +-86, and for gate 16: 846 +-190.

### Changes in Taxonomic distribution after RYGB

Using data from 16S rRNA gene profiling and metaproteomics we were able to describe the taxonomic distribution in microbiota in RYGB and Sham-BWM. The two methods will give differing results since relative abundance data from 16S rRNA gene sequencing describes the community taxonomic structure based on DNA content and therefore the number of cells present, while using data from metaproteomics the analysis is based on on protein content and therefore the more active taxa will be highlighted [[37](#_ENREF_37)].

Metaproteomics revealed that in for RYGB the majority of microbiota protein groups came from *Firmicutes* (ileum content: 34% +- 0.6% SEM,cecum content: 40% +- 1.3% SEM, colon content: 28% +- 1.8% SEM) with *Bacteroidetes* (ileum content: 17% +- 1.7% SEM, cecum content: 12.5% +- 1.4% SEM, colon content: 21.1% +- 3% SEM), *Actinobacteria* (ileum content: 10.8% +- 1% SEM, cecum content: 8,4% +- 1% SEM, colon content: 5.7% +- 1% SEM) and *Proteobacteria* (ileum content: 9.5% +- 0.9% SEM, cecum content: 3.5% +- 0.5% SEM, colon content: 5.8% +- 0.7%) making up significant fractions (Supplemental Figure 3A). Heterogeneous protein groups, those that were assigned to Bacteria but could not be annotated to a phylum, composed a large fraction (ileum content: 28.1% +- 1.7% SEM, cecum content: 35.6% +- 0.6 % SEM, colon content: 38.9% +- 1% SEM) of bacterial protein groups. All other phyla only made up under 1% of microbiota protein group.

For Sham-BWM, metaproteomics revealed that the majority of microbiota protein groups came from *Firmicutes* (ileum content: 51.6% +- 0.6% SEM, cecum content: 52% +- 1.3% SEM, colon content: 38.5% +- 1.5% SEM) with *Bacteroidetes* (ileum content: 5% +- 1% SEM, cecum content: 9.8% +- 0.9% SEM, colon content: 16.5% +- 1.5% SEM), *Actinobacteria* (ileum content: 2.3% +- 0.4% SEM, cecum content: 1.4% +- 0.2%SEM, colon content: 1.9% +- 0.3% SEM) and *Proteobacteria* (ileum content: 5.9% +- 1% SEM, cecum content: 1.9% +- 0.1% SEM, colon content: 1.9% +- 0.3%) making up significant fractions (Supplemental Figure 3A). Heterogeneous protein groups, those that were assigned to Bacteria but could not be annotated to a phylum, composed a large fraction (ileum content: 34.6% +- 0.4% SEM, cecum content: 33.9% +- 0.8 % SEM, colon content: 40.5% +- 0.5% SEM) of bacterial protein groups. All other phyla only made up under 1% of microbiota protein group.

Based on relative number of protein groups, we observed on the genus level that for RYGB some of the most abundant genera were *Clostridium* (ileum content: 5.8% +- 0.8% SEM, cecum content: 4% +- 0.3% SEM, colon content: 3.3% +- 0.1% SEM), *Bifidobacterium* (ileum content: 5.7% +- 0.5% SEM, cecum content: 4.3% +- 0.8% SEM, colon content: 2.6% +- 0.5% SEM), *Prevotella* (ileum content: 1.9% +- 0.2% SEM, cecum content: 1.6% +- 0.2% SEM, colon content: 1.9% +- 0.2% SEM), *Streptococcus* (ileum content: 1.9% +- 0.2% SEM, cecum content: 0.8% +- 0.2% SEM, colon content: 0.7% +- 0.2% SEM), *Bacteroides* (ileum content: 1.9% +- 0.2% SEM, cecum content: 1.3% +- 0.1% SEM, colon content: 1.4% +- 0.3% SEM) and *Lactobacillus* (ileum content: 1.6% +- 0.2% SEM, cecum content: 1.5% +- 0.2% SEM, colon content: 1.1% +- 0.2% SEM) (Supplemental Figure 3B). Some of the most abundant genera in the Sham-BWM samples, based on relative abundance of protein groups, were *Clostridium* (ileum content: 5.4% +- 1.3% SEM, cecum content: 4.2% +- 0.2% SEM, colon content: 3.1% +- 0.3% SEM), *Eubacterium* (ileum content: 0.4% +- 0.1% SEM, cecum content: 2.4% +- 0.5% SEM, colon content: 1.3% +- 0.1% SEM), *Prevotella* (ileum content: 0.6% +- 0.4% SEM, cecum content: 1.4% +- 0.2% SEM, colon content: 2% +- 0.3% SEM), *Streptococcus* (ileum content: 0.8% +- 0.2% SEM, cecum content: 0.2% +- 0.01% SEM, colon content: 0.2% +- 0.03% SEM), *Ruminococcus* (ileum content: 0.3% +- 0.1% SEM, cecum content: 1.3% +- 0.2% SEM, colon content: 1% +- 0.1% SEM) and *Lactobacillus* (ileum content: 13% +- 4.5% SEM, cecum content: 1.7% +- 0.6% SEM, colon content: 2.3% +- 0.4% SEM) (Supplemental Figure 3B).

In 16S rRNA gene profiling all cells are covered including less active and dormant bacterial cells. The taxonomic distribution of bacterial phyla based on this method revealed the same four major phyla as in the metaproteomic analysis, with *Firmicutes* being dominant (Supplemental Figure 4A). In RYGB the distribution of the major phyla was *Firmicutes* (ileum mucus: 84.78% +-3.86% SEM; ileum content: 80.7% +-5.95% SEM; cecum content: 92.78% +-0.69% SEM; colon mucus: 73.9% +-2.35% SEM; colon content: 91.12% +-1.84% SEM), *Actinobacteria* (ileum mucus: 6.71% +-3.58% SEM; ileum content: 14.35% +-6.96% SEM; cecum content: 4.93% +-0.95% SEM; colon mucus: 18.42% +-2.18% SEM; colon content: 4.02% +-1.8% SEM), *Proteobacteria* (ileum mucus: 6.35% +-3.01% SEM; ileum content: 4.56% +-2.34% SEM; cecum content: 1.88% +-0.91% SEM; colon mucus: 4.65% +-0.83% SEM; colon content: 2.48% +-1.21% SEM) and *Bacteroidetes* (ileum mucus: 2.14% +-1.97% SEM; ileum content: 0.38% +-0.29% SEM; cecum content: 0.36% +-0.18% SEM; colon mucus: 2.9% +-0.84% SEM; colon content: 2.33% +-0.92% SEM). In the SHAM-BWM control sample distribution of the major phyla was *Firmicutes* (ileum mucus: 97.62% +-0.54% SEM; ileum content: 98.85% +-0.38% SEM; cecum content: 97.84% +-0.57% SEM; colon mucus: 71.64% +-4.9% SEM; colon content: 85.63% +-1.38% SEM), *Actinobacteria* (ileum mucus: 1.14% +-0.3% SEM; ileum content: 0.64% +-0.09% SEM; cecum content: 0.96% +-0.52% SEM; colon mucus: 24.05% +-4.52% SEM; colon content: 11.61% +-1.71% SEM), *Proteobacteria* (ileum mucus: 0.88% +-0.32% SEM; ileum content: 0.4% +-0.3% SEM; cecum content: 0.83% +-0.2% SEM; colon mucus: 1.95% +-0.27% SEM; colon content: 0.8% +-0.23% SEM) and *Bacteroidetes* (ileum mucus: 0.26% +-0.12% SEM; ileum content: 0.08% +-0.02% SEM; cecum content: 0.02% +-0.01% SEM; colon mucus: 0.74% +-0.08% SEM; colon content: 0.54% +-0.17% SEM).

On the genera taxonomic level, 5 of the most prominent taxa identified in the 16S rRNA gene profiling data of the RYGB sample were *Allobaculum* (ileum mucus: 36.02% +-10.48% SEM; ileum content: 23.13% +-12.42% SEM; cecum content: 10.37% +-2.35% SEM; colon mucus: 30.56% +-5.09% SEM; colon content: 24.3% +-10.86% SEM), *Bifidobacterium* (ileum mucus: 4.39% +-2.27% SEM; ileum content: 3.88% +-2.35% SEM; cecum content: 3.38% +-0.42% SEM; colon mucus: 1.45% +-0.39% SEM; colon content: 2.36% +-0.94% SEM), *Escherichia* (ileum mucus: 5.53% +-2.84% SEM; ileum content: 13.42% +-6.9% SEM; cecum content: 1.64% +-0.76% SEM; colon mucus: 3% +-0.51% SEM; colon content: 2.13% +-0.93% SEM), *Lactobacillus* (ileum mucus: 19.25% +-5.12% SEM; ileum content: 29.92% +-6.7% SEM; cecum content: 11.45% +-1.28% SEM; colon mucus: 15.52% +-4.01% SEM; colon content: 27.14% +-8.47% SEM) and *Streptococcus* (ileum mucus: 5.01% +-1.86% SEM; ileum content: 10.42% +-7.28% SEM; cecum content: 13.61% +-2.84% SEM; colon mucus: 8.8% +-2.47% SEM; colon content: 14.86% +-5.47% SEM) (Supplemental Figure 4B). In the Sham-BWM sample 5 of the most abundant taxa were amongst others, *Allobaculum* (ileum mucus: 28.78% +-14.77% SEM; ileum content: 51.1% +-19.4% SEM; cecum content: 12.04% +-5.24% SEM; colon mucus: 18.78% +-1.47% SEM; colon content: 37.85% +-5.35% SEM), *Clostridium* (ileum mucus: 1.71% +-1.15% SEM; ileum content: 1.33% +-0.68% SEM; cecum content: 22.32% +-2.41% SEM; colon mucus: 2.02% +-0.59% SEM; colon content: 4.36% +-1.83% SEM), *Lactobacillus* (ileum mucus: 41.99% +-18.06% SEM; ileum content: 36.38% +-19.89% SEM; cecum content: 15.52% +-9.27% SEM; colon mucus: 26.04% +-5.02% SEM; colon content: 25.1% +-9% SEM), *Ruminococcus* (ileum mucus: 0.33% +-0.16% SEM; ileum content: 0.09% +-0% SEM; cecum content: 4.3% +-1.18% SEM; colon mucus: 5.79% +-0.75% SEM; colon content: 5.2% +-2.33% SEM) and *Streptococcus* (ileum mucus: 11.28% +-7.55% SEM; ileum content: 2.75% +-0.65% SEM; cecum content: 0.24% +-0.04% SEM; colon mucus: 0.25% +-0.02% SEM; colon content: 0.27% +-0.09% SEM) (Supplemental Figure 4B).

### Protein groups involved in iron utilisation in the ileum after RYGB

Following the metabolome analysis, a metaproteomic analysis of the metabolic pathways was done to identify any changes in protein functions present. In the ileum, a total of 56 protein groups involved in iron uptake and protection were identified as being unique for either RYGB or sham. Of these 55 were unique for RYGB. The protein functions uniquely observed for RYGB were superoxide dismutase (COG0605; 10 protein groups), catalase (COG0753; 3 protein groups), DNA-binding ferritin-like protein (oxidative damage protectant) (COG0783; 1 protein group), ABC-type metal ion transport system, periplasmic component/surface antigen (COG1464; 5 protein groups), ferritin-like protein (COG1528; 5 protein groups), outer membrane receptor proteins, mostly Fe transport (COG1629; 28 protein groups), bacterioferritin (cytochrome b1) (COG2193; 1 protein group), predicted iron-dependent peroxidase (COG2837; 1 protein group) and Mn-containing catalase (COG3546; 1 protein group). Three protein functions significantly increased in relative numbers in RYGB. These were superoxide dismutase (COG0605; *P*<.0001) and two protein functions involved in iron uptake, namely ferritin-like protein (COG1528; *P*=.033) and outer membrane receptor proteins, mostly Fe transport (COG1629; *P*=.029). Most of the protein groups assigned involved in iron uptake and protection were from Bacteroidetes (Supplemental Figure 15A).

### Bacterial xylan degradation and utilisation metabolic pathways are more active in RYGB cecum content

The metaproteomic analysis revealed that protein groups involved in xylan degradation and uptake of resulting degradation products were significantly upregulated in RYGB (Supplemental Figure 15B). Four protein groups assigned to beta-galactosidase/beta-glucuronidase (COG3250) were observed only in RYGB with the relative number of protein groups also significantly higher in RYGB (*P*=.0016). Also, the relative number of protein groups assigned to beta-xylosidase (COG3507; *P*=.012) was significantly higher in RYGB. Three significant α-L-arabinofuranosidases (COG2160) were only identified in RYGB. In addition, the relative number of protein groups assigned to ABC-type xylose transport system, permease component (COG4214; *P*=.0057) was also significantly higher in RYGB. Furthermore, three identified protein groups with the function xylose isomerase (COG2115) exhibited significances difference between RYGB and sham. Two of these were at higher abundance in RYGB while the third was unique for RYGB.

The glycolysis pathway, which also utilises products from the xylose degradation after further conversion by the pentose phosphate pathway, was also seen significantly higher regulated (Supplemental Figure 15C). Most protein groups and functions were more present in RYGB than in sham. The function glyceraldehyde-3-phosphate dehydrogenase (COG0057) was more present in RYGB with all 13 protein groups observed as being significantly more abundant in RYGB. Also, the functions of phosphoglyceromutase (COG0696) and enolase (COG0148) were more strongly represented in RYGB. The former was observed at significantly higher relative numbers in RYGB (*P*=.044), while for the latter all eight quantifiable protein groups with significantly altered abundances in RYGB, were observed with higher abundance.

### Analysis of metabolic pathways of cell sorted bacteria

For metabolic pathways, enrichment was observed for those involved in the biosynthesis of branched-chain and aromatic amino acids as well as histidine metabolism. Folate, pantothenate, porphyrin as well as thiamine functional pathways were also observed enriched for the sorted samples (Supplemental Figure 14). Interestingly the vitamin B6 metabolism and nicotinate metabolism pathways were depleted. Finally, two carbohydrate metabolism functional pathways were also seen as enriched. These were peptidoglycan biosynthesis, and degradation and C5 branched dibasic acid metabolism.

### Functional and taxonomic description of the microbiota detected in the biliopancreatic limb lumen contents

##### Taxonomic analysis

The majority of protein groups identified in the biliopancreatic limb were eukaryotic (74% +-4.3%), with a larger minority originating from bacteria (14.8% +-4.7%). Of the eukaryotic protein groups 89% +-2% could definitively be classed as originating from the host whereas 3.8% +-2.1% originated from feed plants (Supplemental Figure 16A).

The sequencing data revealed that the microbial community inhabiting the biliopancreatic limb had an α-diversity based on Shannon effective of 8.3 +-1.7 and mainly consisted of *Firmicutes* (84.6% +-12.5%), with *Proteobacteria* (11.3 +-14%) and *Actinobacteria* (3.4% +-2.7%) making up substantial minorities (Supplemental Figure 13A). The metaproteomic data also revealed that *Firmicutes* was the dominant phyla (39.8% +-10.9%) with *Actinobacteria* (14.1% +-6.5%) and *Proteobacteria* (13.9% +-6.1%) also making up significant smaller parts of the microbiota, though a large number of bacterial protein groups could not be assigned to a single phylum (21.9% +-4.7%) (Supplemental Figure 13A). In the 16S rRNA gene sequencing data 17 OTU’s were identified with a mean relative number of reads over 0.5% of all reads. Of these, the most abundant OTU was one matched to *Lactobacillus murinus* (22.8% +-14%). Further abundant OTUs were assigned to *Clostridium perfringens*, *Streptococcus hyointestinalis*, and *Bifidobacterium pseudolongum*. These were all at significance altered abundances in the downstream localities of the gut when compared to sham (Supplemental Figure 15B).

##### Functional analysis

In the biliopancreatic limb, metaproteomics revealed that the most abundant functional sub roles in the microbiota were carbohydrate metabolism (22.3% +-7.5%), amino acid metabolism (11% +-3.6%), Translation and ribosomal structure (9% +-5.9%) and Energy production (9.2% +-1%) (Supplemental Figure 13C). Many protein groups from the carbohydrate metabolism were components of ABC-Type sugar transporters (18 protein groups). Bacterial protein groups involved in xylose metabolisms such as ABC-Type transporters for xylose (2 protein groups), beta-xylosidase (2 protein groups) and xylose isomerase (2 protein groups) were also identified. For the functional sub-role of amino acid metabolism, a number of protein groups involved in uptake and degradation of proteins were identified. These included ABC-type transporters for amino acid or peptides (12 protein groups) and peptidases (12 protein groups) (Supplemental Figure 16C).

## Supplemental Discussion

In this study, we investigated the adaptation of the intestinal microbiota to RYGB. This procedure leads to striking changes in the physical and chemical environment present in the gastrointestinal tract. These include a change in pH, different oxygen levels, different composition of the nutrients and alterations in bile acid concentrations which are known to inhibit bacteria growth [[38](#_ENREF_38), [39](#_ENREF_39), [40](#_ENREF_40), [41](#_ENREF_41)]. Thus, these factors should have a considerable influence on the composition of the microbiota after RYGB. Bacteria are known to generally have very malleable metabolisms which can be tailored to suit different environmental influences. This means that not only is the study of which bacteria taxa are present and how their distribution changes in the microbiota of interest, but more importantly is how they functionally adapt to environmental changes. By applying a comprehensive multi-omics approach, we were able to investigate not only taxonomic changes but also functional changes in the microbial community. On a global view, the results suggested profound changes in the microbiota on a taxonomic as well as functional level. In a previous study, metaproteomic taxa data was found to accurately describe the biomass changes, especially of the proteogenous biomass, whereas 16S rRNA gene sequencing data reveals changes in the cell numbers [[42](#_ENREF_42)]. Thereby metaproteomics, although usually with a lower resolution for taxa identification than 16S rRNA gene sequencing, would reveal changes in the metabolically active part of the intestinal microbiota. 16S rRNA gene sequencing was able to describe all bacteria found in the gut, including the dormant and less active taxa.

### Functional changes in the microbiota are more localised in the gut

Most iron is taken up by the host in the duodenum [[43](#_ENREF_43), [44](#_ENREF_44)]. Since after RYGB the ingested nutrients do not pass the duodenum after the anatomical rearrangement of the gastrointestinal tract, iron uptake by the host is reduced [[45](#_ENREF_45)]. Hence, iron should be present at higher abundances in the gut lumen of RYGB. This could explain the greater number of bacterial protein groups involved in protection from iron identified in the ileum after RYGB. Superoxidase, catalase and Ferritin-like protein are known to protect from iron or prevent harmful reactive oxidative species produced by reactions with iron [[46](#_ENREF_46), [47](#_ENREF_47)]. It was observed in the ileum that for Bacteroidetes the relative number of protein groups identified which were involved in iron metabolism increased in RYGB.

### Xylan metabolism altered in the microbiota

Functional alterations in the cecum were observed with an increase in xylan and xylose degradation protein groups. These results fit well with the literature wherein a rat model after RYGB there was a decrease in xylose absorption into the host [[48](#_ENREF_48)]. Our data suggest that for the cecum the higher observed abundances and numbers of protein groups involved in the utilisation of xylose could be due to that xylan is being utilised to a greater extent in RYGB by the microbiota, therefore being less available for the host. The increase in *Bifidobacterium* which are known xylo-oligosaccharide utilizers would underline this [[49](#_ENREF_49)]. In addition, the observation of a greater abundance of *Bacteroidetes* and more specifically *Bacteroideceae*, these are also well known as xylan degraders, strengthens this assumption [[50](#_ENREF_50), [51](#_ENREF_51)].

### Species found at high abundance in the gut after RYGB are probably seeded from the microbiota observed in the biliopancreatic limb

A number of taxa which were observed at high abundances in the biliopancreatic limb were also seen downstream in the distal gut at significant higher abundances in RYGB. The significant higher abundance of *Clostridium perfringens* in the cecum, as well as the colon content, was especially interesting because although this species is naturally found at low abundances in the gut of healthy individuals, after proliferation can become pathogenic [[52](#_ENREF_52)]. It is known to cause histotoxic and intestinal infections [[53](#_ENREF_53)]. It can disrupt the tight junctions causing leakage of the epithelium in the gut, and its repertoire of toxins can greatly affect the host, and even cause death [[54](#_ENREF_54), [55](#_ENREF_55), [56](#_ENREF_56)]. The higher levels of *Clostridium perfringens* after RYGB are a cause for concern. *Streptococcus hyointestinalis* was also detected at high abundances in the biliopancreatic limb as well as at significantly higher abundances in multiple distal regions of the intestinal tract in RYGB. Strains from this species are known to produce Bacteriocins which are generally thought to be probiotic by helping control pathogenic bacteria [[57](#_ENREF_57)]. *Bifidobacterium pseudolongum,* which was also abundant in the biliopancreatic limb, was observed at significantly higher abundance in the ileum and cecum for RYGB. Possibly for these species, the microbial community in biliopancreatic limb continually seeds the rest of the gut with these microbes maybe causing them to inhabit the gut at higher abundances.

## Supplemental References

1 Seyfried F, Miras AD, Bueter M, Prechtl CG, Spector AC, le Roux CW. Effects of preoperative exposure to a high-fat versus a low-fat diet on ingestive behavior after gastric bypass surgery in rats. Surg Endosc 2013;**27**:4192-201.

2 Seyfried F, Bueter M, Spliethoff K, Miras AD, Abegg K, Lutz TA*, et al.* Roux-en Y gastric bypass is superior to duodeno-jejunal bypass in improving glycaemic control in Zucker diabetic fatty rats. Obes Surg 2014;**24**:1888-95.

3 Jehmlich N, Hubschmann T, Gesell Salazar M, Volker U, Benndorf D, Muller S*, et al.* Advanced tool for characterization of microbial cultures by combining cytomics and proteomics. Appl Microbiol Biotechnol 2010;**88**:575-84.

4 Caporaso JG, Kuczynski J, Stombaugh J, Bittinger K, Bushman FD, Costello EK*, et al.* QIIME allows analysis of high-throughput community sequencing data. Nat Methods 2010;**7**:335-6.

5 Edgar RC. Search and clustering orders of magnitude faster than BLAST. Bioinformatics 2010;**26**:2460-1.

6 Dowd SE, Callaway TR, Wolcott RD, Sun Y, McKeehan T, Hagevoort RG*, et al.* Evaluation of the bacterial diversity in the feces of cattle using 16S rDNA bacterial tag-encoded FLX amplicon pyrosequencing (bTEFAP). BMC microbiology 2008;**8**:125.

7 Eren AM, Zozaya M, Taylor CM, Dowd SE, Martin DH, Ferris MJ. Exploring the diversity of Gardnerella vaginalis in the genitourinary tract microbiota of monogamous couples through subtle nucleotide variation. PloS one 2011;**6**:e26732.

8 Dowd SE, Sun Y, Wolcott RD, Domingo A, Carroll JA. Bacterial tag-encoded FLX amplicon pyrosequencing (bTEFAP) for microbiome studies: bacterial diversity in the ileum of newly weaned Salmonella-infected pigs. Foodborne pathogens and disease 2008;**5**:459-72.

9 Capone KA, Dowd SE, Stamatas GN, Nikolovski J. Diversity of the human skin microbiome early in life. J Invest Dermatol 2011;**131**:2026-32.

10 Swanson KS, Dowd SE, Suchodolski JS, Middelbos IS, Vester BM, Barry KA*, et al.* Phylogenetic and gene-centric metagenomics of the canine intestinal microbiome reveals similarities with humans and mice. The ISME journal 2011;**5**:639-49.

11 Nguyen N-P, Warnow T, Pop M, White B. A perspective on 16S rRNA operational taxonomic unit clustering using sequence similarity. npj Biofilms and Microbiomes 2016;**2**:16004.

12 DeSantis TZ, Hugenholtz P, Larsen N, Rojas M, Brodie EL, Keller K*, et al.* Greengenes, a chimera-checked 16S rRNA gene database and workbench compatible with ARB. Appl Environ Microbiol 2006;**72**:5069-72.

13 Cole JR, Wang Q, Fish JA, Chai B, McGarrell DM, Sun Y*, et al.* Ribosomal Database Project: data and tools for high throughput rRNA analysis. Nucleic Acids Res 2014;**42**:D633-42.

14 Haange SB, Jehmlich N, Hoffmann M, Weber K, Lehmann J, von Bergen M*, et al.* Disease Development Is Accompanied by Changes in Bacterial Protein Abundance and Functions in a Refined Model of Dextran Sulfate Sodium (DSS)-Induced Colitis. J Proteome Res 2019;**18**:1774-86.

15 Lagkouvardos I, Fischer S, Kumar N, Clavel T. Rhea: a transparent and modular R pipeline for microbial profiling based on 16S rRNA gene amplicons. Peerj 2017;**5**:e2836.

16 Gallagher SR. One-dimensional SDS gel electrophoresis of proteins. Current protocols in protein science / editorial board, John E Coligan [et al] 2012;**Chapter 10**:Unit 10 1 1-44.

17 Perez-Riverol Y, Csordas A, Bai J, Bernal-Llinares M, Hewapathirana S, Kundu DJ*, et al.* The PRIDE database and related tools and resources in 2019: improving support for quantification data. Nucleic Acids Res 2019;**47**:D442-D50.

18 Schneider T, Schmid E, de Castro JV, Jr., Cardinale M, Eberl L, Grube M*, et al.* Structure and function of the symbiosis partners of the lung lichen (Lobaria pulmonaria L. Hoffm.) analyzed by metaproteomics. Proteomics 2011;**11**:2752-6.

19 Tatusov RL, Natale DA, Garkavtsev IV, Tatusova TA, Shankavaram UT, Rao BS*, et al.* The COG database: new developments in phylogenetic classification of proteins from complete genomes. Nucleic Acids Res 2001;**29**:22-8.

20 Koskinen VR, Emery PA, Creasy DM, Cottrell JS. Hierarchical clustering of shotgun proteomics data. Mol Cell Proteomics 2011;**10**:M110 003822.

21 Benjamini Y, Hochberg Y. Controlling the False Discovery Rate - a Practical and Powerful Approach to Multiple Testing. J Roy Stat Soc B Met 1995;**57**:289-300.

22 Kanehisa M, Goto S. KEGG: kyoto encyclopedia of genes and genomes. Nucleic Acids Res 2000;**28**:27-30.

23 Caspi R, Billington R, Ferrer L, Foerster H, Fulcher CA, Keseler IM*, et al.* The MetaCyc database of metabolic pathways and enzymes and the BioCyc collection of pathway/genome databases. Nucleic Acids Res 2016;**44**:D471-80.

24 Becker BJ. Combining significance levels. New York: Russell Sage Foundation, 1994.

25 Pham HT, Arnhard K, Asad YJ, Deng L, Felder TK, St. John-Williams L*, et al.* Inter-Laboratory Robustness of Next-Generation Bile Acid Study in Mice and Humans: International Ring Trial Involving 12 Laboratories. The Journal of Applied Laboratory Medicine: An AACC Publication 2016.

26 Siskos AP, Jain P, Romisch-Margl W, Bennett M, Achaintre D, Asad Y*, et al.* Interlaboratory Reproducibility of a Targeted Metabolomics Platform for Analysis of Human Serum and Plasma. Anal Chem 2017;**89**:656-65.

27 Wissenbach DK, Oliphant K, Rolle-Kampczyk U, Yen S, Hoke H, Baumann S*, et al.* Optimization of metabolomics of defined in vitro gut microbial ecosystems. International journal of medical microbiology : IJMM 2016;**306**:280-9.

28 Ivanisevic J, Zhu ZJ, Plate L, Tautenhahn R, Chen S, O'Brien PJ*, et al.* Toward 'omic scale metabolite profiling: a dual separation-mass spectrometry approach for coverage of lipid and central carbon metabolism. Anal Chem 2013;**85**:6876-84.

29 Samino S, Vinaixa M, Diaz M, Beltran A, Rodriguez MA, Mallol R*, et al.* Metabolomics reveals impaired maturation of HDL particles in adolescents with hyperinsulinaemic androgen excess. Scientific reports 2015;**5**:11496.

30 Gowda H, Ivanisevic J, Johnson CH, Kurczy ME, Benton HP, Rinehart D*, et al.* Interactive XCMS Online: Simplifying Advanced Metabolomic Data Processing and Subsequent Statistical Analyses. Anal Chem 2014;**86**:6931-9.

31 Friedman J, Alm EJ. Inferring correlation networks from genomic survey data. PLoS Comput Biol 2012;**8**:e1002687.

32 Shannon P, Markiel A, Ozier O, Baliga NS, Wang JT, Ramage D*, et al.* Cytoscape: a software environment for integrated models of biomolecular interaction networks. Genome Res 2003;**13**:2498-504.

33 Faust K, Raes J. CoNet app: inference of biological association networks using Cytoscape. F1000Research 2016;**5**:1519.

34 Brown MB. 400: A Method for Combining Non-Independent, One-Sided Tests of Significance. Biometrics 1975;**31**:987-92.

35 Su G, Kuchinsky A, Morris JH, States DJ, Meng F. GLay: community structure analysis of biological networks. Bioinformatics 2010;**26**:3135-7.

36 Guimera R, Nunes Amaral LA. Functional cartography of complex metabolic networks. Nature 2005;**433**:895-900.

37 Kleiner M, Thorson E, Sharp CE, Dong X, Liu D, Li C*, et al.* Assessing species biomass contributions in microbial communities via metaproteomics. Nature communications 2017;**8**:1558.

38 Zhang H, DiBaise JK, Zuccolo A, Kudrna D, Braidotti M, Yu Y*, et al.* Human gut microbiota in obesity and after gastric bypass. Proc Natl Acad Sci U S A 2009;**106**:2365-70.

39 Bhutta HY, Rajpal N, White W, Freudenberg JM, Liu Y, Way J*, et al.* Effect of Roux-en-Y gastric bypass surgery on bile acid metabolism in normal and obese diabetic rats. PLoS One 2015;**10**:e0122273.

40 Daniel H, Gholami AM, Berry D, Desmarchelier C, Hahne H, Loh G*, et al.* High-fat diet alters gut microbiota physiology in mice. Isme J 2014;**8**:295-308.

41 Devkota S, Wang Y, Musch MW, Leone V, Fehlner-Peach H, Nadimpalli A*, et al.* Dietary-fat-induced taurocholic acid promotes pathobiont expansion and colitis in Il10-/- mice. Nature 2012;**487**:104-8.

42 Kleiner P, Heydenreuter W, Stahl M, Korotkov VS, Sieber SA. A Whole Proteome Inventory of Background Photocrosslinker Binding. Angew Chem Int Ed Engl 2017;**56**:1396-401.

43 Kortman GA, Raffatellu M, Swinkels DW, Tjalsma H. Nutritional iron turned inside out: intestinal stress from a gut microbial perspective. FEMS Microbiol Rev 2014;**38**:1202-34.

44 Leung PS, Srai SK, Mascarenhas M, Churchill LJ, Debnam ES. Increased duodenal iron uptake and transfer in a rat model of chronic hypoxia is accompanied by reduced hepcidin expression. Gut 2005;**54**:1391-5.

45 Gesquiere I, Lannoo M, Augustijns P, Matthys C, Van der Schueren B, Foulon V. Iron deficiency after Roux-en-Y gastric bypass: insufficient iron absorption from oral iron supplements. Obes Surg 2014;**24**:56-61.

46 Smith JL. The physiological role of ferritin-like compounds in bacteria. Critical reviews in microbiology 2004;**30**:173-85.

47 Wessling-Resnick M. Biochemistry of Iron Uptake. Critical Reviews in Biochemistry and Molecular Biology 1999;**34**:285-314.

48 Guijarro A, Suzuki S, Chen C, Kirchner H, Middleton FA, Nadtochiy S*, et al.* Characterization of weight loss and weight regain mechanisms after Roux-en-Y gastric bypass in rats. American Journal of Physiology - Regulatory, Integrative and Comparative Physiology 2007;**293**:R1474-R89.

49 Christensen EG, Licht TR, Leser TD, Bahl MI. Dietary Xylo-oligosaccharide stimulates intestinal bifidobacteria and lactobacilli but has limited effect on intestinal integrity in rats. BMC Res Notes 2014;**7**:660.

50 Dodd D, Mackie RI, Cann IK. Xylan degradation, a metabolic property shared by rumen and human colonic Bacteroidetes. Mol Microbiol 2011;**79**:292-304.

51 Zhang ML, Chekan JR, Dodd D, Hong PY, Radlinski L, Revindran V*, et al.* Xylan utilization in human gut commensal bacteria is orchestrated by unique modular organization of polysaccharide-degrading enzymes. Proc Natl Acad Sci U S A 2014;**111**:E3708-E17.

52 Finegold SM, Summanen PH, Downes J, Corbett K, Komoriya T. Detection of Clostridium perfringens toxin genes in the gut microbiota of autistic children. Anaerobe 2017;**45**:133-7.

53 Galili T, Gingold H, Shaul S, Benjamini Y. Identifying the ligated amino acid of archaeal tRNAs based on positions outside the anticodon. Rna 2016;**22**:1477-91.

54 Eichner M, Protze J, Piontek A, Krause G, Piontek J. Targeting and alteration of tight junctions by bacteria and their virulence factors such as Clostridium perfringens enterotoxin. Pflugers Arch 2017;**469**:77-90.

55 Mitchell LA, Koval M. Specificity of Interaction between Clostridium perfringens Enterotoxin and Claudin-Family Tight Junction Proteins. Toxins 2010;**2**:1595-611.

56 Uzal FA, Freedman JC, Shrestha A, Theoret JR, Garcia J, Awad MM*, et al.* Towards an understanding of the role of Clostridium perfringens toxins in human and animal disease. Future Microbiol 2014;**9**:361-77.

57 Dobson A, Cotter PD, Ross RP, Hill C. Bacteriocin production: a probiotic trait? Appl Environ Microbiol 2012;**78**:1-6.
